# Abundance of microplastics and nanoplastics in urban atmosphere

**DOI:** 10.1126/sciadv.adz7779

**Published:** 2026-01-07

**Authors:** Tafeng Hu, Chongchong Zhang, Yuqing Zhu, Jing Duan, Suixin Liu, Niu Jin, Yingpan Song, Feng Wu, Jianjun Li, Ting Zhang, Hongya Niu, Xuxiang Li, Hong Huang, Gary S. Casuccio, Yu Huang, Kin-Fai Ho, Junji Cao, Daizhou Zhang

**Affiliations:** ^1^ State Key Laboratory of Loess Science, National Observation and Research Station of Regional Ecological Environment Change and Comprehensive Management in the Guanzhong Plain, Institute of Earth Environment, Chinese Academy of Sciences, Xi’an 710061, China.; ^2^ Nanjing University of Information Science and Technology, Nanjing 210044, China.; ^3^ School of Earth Science and Engineering, Hebei University of Engineering, Handan 056038, Hebei, China.; ^4^ Xi’an Jiaotong University, Xi’an 710049, China.; ^5^ School of Resources and Environment, Nanchang University, Nanchang 330031, China.; ^6^ RJ Lee Group Inc., Pittsburgh, PA 15239, USA.; ^7^ The Jockey Club School of Public Health and Primary Care, The Chinese University of Hong Kong, Shatin, N.T., Hong Kong SAR 999077, China.; ^8^ Institute of Atmospheric Physics, Chinese Academy of Sciences, Beijing 100029, China.; ^9^ Faculty of Environmental and Symbiotic Sciences, Prefectural University of Kumamoto, Kumamoto 862-8502, Japan.

## Abstract

Microplastics (MPs) and nanoplastics (NPs) are emerging environmental pollutants, yet their behavior in the atmosphere remains poorly understood. Using an innovative method capable of detecting plastic particles as small as 200 nanometers, we quantified MPs and NPs in aerosols, dry and wet deposition, and resuspension in two Chinese megacities, Guangzhou and Xi’an. Airborne concentrations reached 1.8 × 10^5^ MPs per cubic meter and 5.0 × 10^4^ NPs per cubic meter in Guangzhou and 1.4 × 10^5^ MPs per cubic meter and 3.0 × 10^4^ NPs per cubic meter in Xi’an. Estimates revealed a variation of two to five orders of magnitude in MP and NP fluxes across major atmospheric compartments, dominated by road dust resuspension and rainfall-driven wet precipitation. Plastic particles were more heterogeneously mixed in deposition samples than in aerosols and resuspension, indicating enhanced aggregation and removal. These results provide an integrated assessment of MPs and NPs in urban atmospheric processes and offer critical insights into their transformation, fate, and potential implication for climate, ecosystems, and human health.

## Teaser

A semiautomated microanalysis approach to quantify atmospheric plastics and their cross-compartmental flux in two megacities.

## INTRODUCTION

Over the past two decades, plastic as an emerging pollutant has become a global issue because of its widespread distribution in every environmental compartment of the Earth system (*1*). Originating from urban, agricultural, and industrial sources, larger waste plastic items undergo fragmentation via physical, chemical, or biological processes over time and generate numerous smaller plastic debris or particles (*2*, *3*). Those fragmentation of synthetic or naturally modified polymers produced microplastics (MPs; 1 μm to 5 mm) (*4*) and nanoplastics (NPs; <1 μm) (*5*, *6*). Similar to MPs, NP particles are present in the environment globally and cover a large and continuous spectrum of sizes and shapes including fibers, film fragments, spherules, and foam pellets (*7*). Related to their nanometric size range and heterogeneity, study on NPs frequently reveals distinct environmental implications and usually faces analytical challenges (*8*, *9*).

MPs and NPs have been found not only in cities but also in remote polar and high-altitude regions (*10*, *11*), and transport by wind at transcontinental and transoceanic scales was suggested as an important route for the spread of plastic in the global carbon cycle (*12*, *13*). Hence, observational data on emission, distribution, and deposition of atmospheric MPs and NPs are vital in the quantitative assessment of plastic burden and their fluxes within Earth’s three physical compartments, i.e., land, atmosphere, and water (*14*). Density, size, shape, and processing of atmospheric plastics may be like aerosol particles that can influence particle transport and Earth’s energy balance via direct radiative effects (*15*). MPs have been modeled to cause positive and negative radiative forcing depending on their size, morphology, and vertical distribution (*16*). Because of their hydrophobic nature, atmospheric plastics were often regarded as inactive cloud condensation nuclei and active ice-nucleating particles. Intriguingly, physical and chemical changes on surface defect, roughness, and mixing states of plastics through weathering and atmospheric aging processes favor both their cloud condensation nuclei (*17*, *18*) and ice-nucleating (*19*, *20*) abilities.

Airborne plastics pose substantial health risks because of their rapid atmospheric transport (*21*). Although the actual effects of environmental plastic particles are insufficiently understood (*1*), dose-dependent effects or diseases of micrometer-sized versus nanometer-sized particles on biota and humans were mostly related to inhalation (*22*). Once inhaled into the human body, both MPs and NPs are believed to release their constituents, sorbed species, and pathogenic organisms (*23*). Knowledge of the chemical and microphysical properties of plastic particles in the atmosphere is crucial to assess their health impacts (*24*). Size, shape, and specific surface area are proposed as possible drivers for MPs and NPs’ toxicity (*25*).

Because of the limitations of current quantification techniques, reported plastic concentrations in environmental samples are likely substantially underestimated (*13*, *15*–*17*). The analytical methods for plastic particles typically consist of three main steps: (i) plastic identification, (ii) quantification (either by particle counting or mass measurement), and (iii) characterization of physicochemical properties. However, technical challenges remain in both the identification and quantification of atmospheric plastics, primarily due to interference from impurities within complex environmental matrices. Consequently, separation techniques based on density or size, along with digestion pretreatments, are also essential for isolating and purifying plastic particles (*26*, *27*). To date, prevailing methods for identifying MPs and NPs fall into two categories: bulk analysis and single-particle analysis, each involving trade-offs between spatial resolution and sensitivity. Bulk analysis techniques, such as mass spectrometry or thermal analysis, measure the total mass rather than the number abundance of plastic particles. This requires prior isolation of specific size fractions through filtration or size fractionation (*26*, *28*). Although highly sensitive for detecting low NP masses (down to picograms) (*18*, *29*), bulk methods cannot provide particle counts, size distributions, or morphological data. Single-particle analysis uses optical or electron microscopy to identify particles on the basis of physical traits (e.g., morphology, color, and transparency), followed by polymer confirmation via vibrational spectroscopy (e.g., infrared or Raman). This approach allows discrete particle counting but is limited by optical wavelength, restricting applicability to plastics larger than ~1 μm (*30*, *31*). Recent advances claim improved spatial resolutions from 500 nm with signal enhancement to 100 nm using image processing or machine learning (*9*, *27*, *32*–*37*). However, most achievements use pristine reference materials or clean simulated samples, with no validation from environmental samples. Moreover, this method only targets suspected particles, and reliance on nonautomated visual inspection introduces human bias, compromising statistical significance and yielding unquantifiable results (*7*, *38*–*39*). As an alternative that avoids errors introduced by human operators, an automated technique known as computer-controlled scanning electron microscopy (CCSEM) is emerging. This technique minimizes user bias by automating the selection of particles of interest, thereby allowing the analysis of up to hundreds of particles within an hour (*40*).

At present, the integration of plastic particles into atmospheric transport and chemistry models remains limited because of a lack of sufficient observational data. In particular, there is a notable scarcity of field data concerning the cross-compartment transport of MPs and NPs, as well as on their size-resolved abundances and morphologies (*16*, *41*). To address this gap, we developed a semiautomated microanalytical approach capable of identifying individual plastic particles down to 200 nm in size. Using a CCSEM system equipped with energy-dispersive x-ray spectroscopy (EDX), we located and measured individual particles in environmental samples collected from Guangzhou (GZ; a coastal megacity in southern China) and Xi’an (XA, an inland megacity in northwestern China). Subsequent manual relocation and identification enabled the determination of number concentrations and physicochemical characteristics of MPs and NPs across aerosols, dry and wet deposition, and resuspended road dust. These size-resolved data on atmospheric plastic particles may offer insights into their effects on ecosystems and human health, as well as their influence on Earth’s energy balance.

## RESULTS

### Abundances and flux estimates of MPs and NPs

**Fig. 1.**
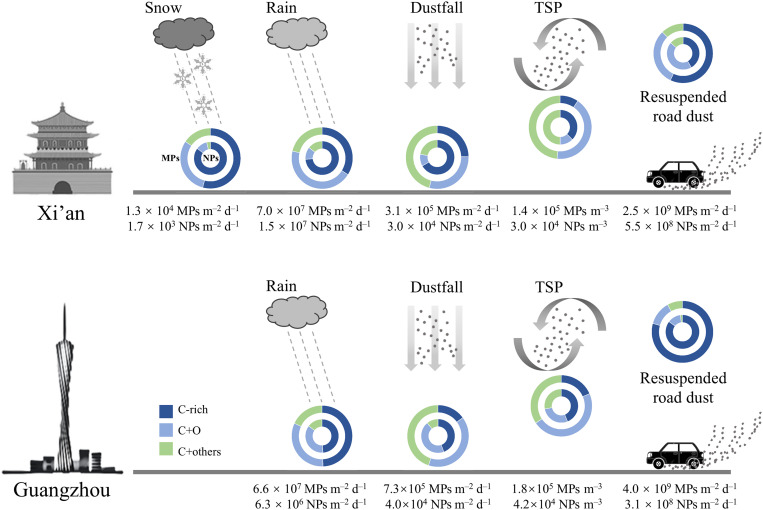
MP and NP abundances in aerosols and estimated fluxes across atmospheric compartments in semiarid (XA) and humid subtropical (GZ) urban environments. Ring slices illustrate the polymer composition with the number fractions of C-rich, C + O (C + O–rich), and C + others polymer species in each atmospheric MP (outer) and NP (inner) group. Plastic fluxes are based on single sampling events, whereas the TSP data are derived from 5-day composite samples. d, day.

Abundances of airborne plastic particles and their fluxes across various compartments of the atmospheric system in GZ and XA are presented in Fig. 1. The order-of-magnitude flux estimates were obtained from measured plastic particle counts, sampling parameters, and meteorological records, following the procedures detailed in Materials and Methods. In each environmental sample, the abundance of MP particles exceeded that of NPs. The number concentrations of MPs and NPs in total suspended particles (TSPs) were measured at 1.8 × 10^5^ m^−3^ and 4.2 × 10^4^ m^−3^ in GZ and 1.4 × 10^5^ m^−3^ and 3.0 × 10^4^ m^−3^ in XA, respectively. The number concentrations of plastic particles in rainwater from GZ were measured at 6.0 × 10^6^ MPs liter^−1^ and 5.7 × 10^5^ NPs liter^−1^. In XA, comparable concentrations were observed in rainwater (2.0 × 10^6^ MPs liter^−1^ and 4.2 × 10^5^ NPs liter^−1^) and snowmelt (2.1 × 10^6^ MPs liter^−1^ and 2.7 × 10^5^ NPs liter^−1^). Although the abundances of MPs and NPs in rainwater were higher in GZ than in XA, the greater precipitation rate recorded during the sampling period in XA (as provided in Materials and Methods) led to a higher wet deposition flux via rainfall, reaching 7.0 × 10^7^ MPs m^−2^ day^−1^ and 1.5 × 10^7^ NPs m^−2^ day^−1^. The dry deposition fluxes were measured at 7.3 × 10^5^ MPs m^−2^ day^−1^ and 4.0 × 10^4^ NPs m^−2^ day^−1^ in GZ and 3.1 × 10^5^ MPs m^−2^ day^−1^ and 3.0 × 10^4^ NPs m^−2^ day^−1^ in XA. These results indicate that wet deposition via rainfall was the dominant removal pathway for plastic particles from the urban atmosphere. In road dust resuspension samplings, where a friction velocity of 0.54 m s^−1^ was applied to simulate dust mobilization from surface soils, the number concentrations of plastic particles reached 2.8 × 10^6^ MPs m^−3^ and 2.2 × 10^6^ NPs m^−3^ in GZ compared to 1.8 × 10^6^ MPs m^−3^ and 3.9 × 10^5^ NPs m^−3^ in XA. On the basis of the bottom area of the resuspension chamber and the airflow rate of the exhaust, the estimated emission fluxes from road dust resuspension were 4.0 × 10^9^ MPs m^−2^ day^−1^ and 3.1 × 10^8^ NPs m^−2^ day^−1^ in GZ and 2.5 × 10^9^ MPs m^−2^ day^−1^ and 5.5 × 10^8^ NPs m^−2^ day^−1^ in XA.

Our semiautomated microanalysis divided the plastic particles, according to their elemental compositions, into three subgroups, including C-rich, C + O–rich (called C + O), and C + others particles. EDX analysis showed that the spectra of typical MPs in these subgroups matched those of their corresponding reference materials (fig. S1). Representative morphologies and elemental compositions of detected NPs are depicted in fig. S2. The C-rich group represents hydrocarbon polymers such as polyethylene (PE), polypropylene (PP), polystyrene (PS), and synthetic rubber. Particles in the C + O group are C- and O-containing polymers, commonly known as polyethylene terephthalate (PET), polycarbonate (PC), polyvinyl alcohol (PVA), polymethyl methacrylate (PMMA), epoxy resin, etc. Particles in the C + others group also contain elemental F, Cl, N, etc., indicating polyvinyl chloride (PVC), polytetrafluoroethene (PTFE or Teflon), polyurethane (PU), polyamide (PA or Nylon), and other additives. Ring charts in Fig. 1 illustrate the relative abundances of C-rich, C + O, and C + others polymer species in each atmospheric MP and NP group.

The most abundant MP and NP particles were in the resuspended road dust samples in both cities, occupying 6.70 and 4.03% (in blank-subtracted numbers) of total particles in respective micro- and nanosize ranges in GZ and 3.41 and 1.92% of those in XA. The lowest plastic particle number abundances occurred in the TSP samples, accounting for 2.36% (MPs in total microscale particles) and 0.48% (NPs in total nanoscale particles) in GZ and 1.75% (MPs) and 0.47% (NPs) in XA. As for polymer species, no obvious pattern of C-rich, C + O, and C + others subgroup number percentages in MPs and NPs can be seen among different atmospheric samples and between the two cities.

### Size distribution and morphological factors

**Fig. 2.**
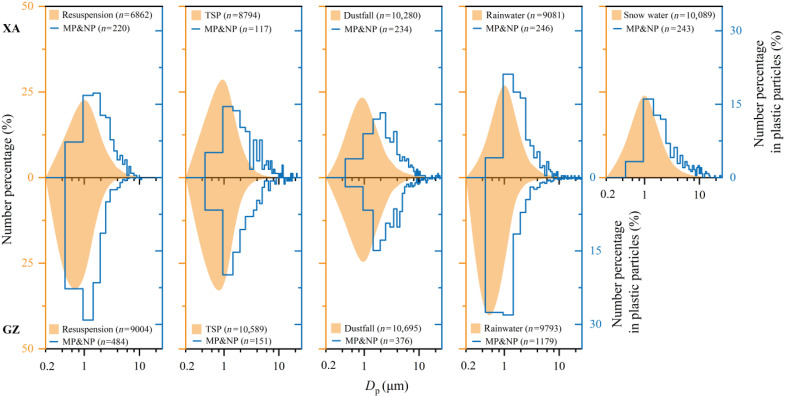
Size-resolved abundance of atmospheric MPs and NPs across urban compartments in XA and GZ. Particle number distributions show relative abundance stratified by size class. The numerical values in parentheses for each sample correspond to the total particle counts (orange-filled curves) and the plastic-only counts (blue lines). The total particle count encompasses both plastic and nonplastic particles.

The size distribution and morphological characteristics of MPs and NPs among the atmospheric particles in different environmental samples collected from XA and GZ city are illustrated in Fig. 2 and figs. S3 to S5. Most of the MP and NP particles showed monomodal patterns of size distribution in their respective micro- and nanosize ranges, with all the size peaks in the range of 1.0 to 2.0 μm (Fig. 2). Visual identification of MPs in conventional analytical methods relies on recognizing the fibrous shape, color, and transparency of plastic particles under a microscope. As a result, fibers have been the most frequently reported type of MP. In contrast, our semiautomated microanalysis identifies plastic particles on the basis of their carbon-containing elemental signature. This approach revealed that plastic fibers (with their aspect ratio reaching 3) account for less than 5% (in numbers) of total MPs and NPs in all the samples (fig. S3). More round (fig. S4) atmospheric MPs and NPs with rough (fig. S5) surfaces are found in GZ than those in XA.

The maximum and minimum diameters of plastic particle with their average size in each atmospheric sample are listed in Table 1. The smallest NP particle appeared in resuspension samples at both cities, including a 266-nm particle in XA and a 289-nm NP in GZ. The largest MP particle in XA was a TSP particle, with a diameter of 62.0 μm, while the maximum MP size of 52.2 μm in GZ was observed in the rainwater sample. The maximum, minimum, and average diameters of plastic particles were dispersed across all types of environmental samples in the two cities studied.

**Table 1.** Size ranges and average diameters of atmospheric MPs and NPs in urban compartments in XA and GZ. Note: *N*_MPs_ is the particle number of MPs. *N*_totM_ is the total number of micrometer-sized particles. *N*_NPs_ is the particle number of NPs. *N*_totN_ is the total number of nanosized particle. n.a. means not available.

			Resuspension	TSP	Dustfall	Rainwater	Snow water
XA	MPs	*N*_MPs_/*N*_totM_	183/4989	101/5414	216/7324	204/6554	216/7437
		Max. (μm)	9.9	62	25.9	39.1	28.9
		*D*_p_ ± SD (μm)	2.9 ± 1.7	5.3 ± 7.6	4.4 ± 4.2	3.7 ± 4.8	5.5 ± 5.1
	NPs	*N*_NPs_/*N*_totN_	37/1873	16/3380	18/2956	42/2527	27/2652
		Min. (nm)	266	529	354	484	563
		*D*_p_ ± SD (nm)	726 ± 171	744 ± 128	649 ± 174	809 ± 121	804 ± 136
GZ	MPs	*N*_MPs_/*N*_totM_	279/3912	126/4950	358/7375	611/2634	n.a.
		Max. (μm)	27.7	26.6	24	52.2	n.a.
		*D*_p_ ± SD (μm)	2.1 ± 2.0	4.0 ± 4.6	3.9 ± 3.1	5.4 ± 7.5	n.a.
	NPs	*N*_NPs_/*N*_totN_	205/5092	25/5639	18/3320	568/7159	n.a.
		Min. (nm)	289	440	489	386	n.a.
		*D*_p_ ± SD (nm)	669 ± 181	732 ± 152	771 ± 155	680 ± 158	n.a.

## DISCUSSION

### More atmospheric plastic than expected

Once studied as marine pollutants, MPs have now been detected in soils, biota, and atmosphere. Some geoscientists have considered plastics as emerging geomaterials with their specific chemistries not previously seen in Earth’s history (*14*). As a component of the global carbon cycle, the stocks, sources, transformations of plastic, and their fates are of geochemical and societal significance (*42*, *43*). Thus, growing concern over plastic contamination, originally centered on environmental effects, has now engaged the scientific community of Earth’s biogeochemical cycles and climate change. Both fields face analytical challenges in detecting and characterizing these incidental materials across scales from micro- to nanosized particles (*1*, *13*, *44*). The lack of plastic quantification in the air has resulted in numerous assumptions and uncertainties in the global modeling of plastic cycle processes (*16*). An exact magnitude of plastics in the atmosphere may affect the material and energy balance of a layer enveloping the entire planet, with potential ecological and biogeochemical consequences across terrestrial and marine systems.

By quantifying all thousands of individual particles on a CCSEM substrate, our semiautomated microanalysis is less prone to human bias than conventional plastic identification through manual inspection. These quantitative results showed substantial amounts of plastic particles in all the environmental samples. The plastic particle number concentrations in TSP and dustfall fluxes (Fig. 1) are 10^2^ to 10^6^ times greater than values obtained through visual identification techniques [i.e., manually operated scanning electron microscopy with EDX (SEM-EDX), micro–Fourier transform infrared spectrometry, or μ-Raman] (*15*, *16*, *45*). Even with a size detection limit of 200 nm, our CCSEM system measured plastic concentrations in environmental samples (rainwater and snowmelt; Fig. 1) that were one order of magnitude higher than those in bottled water analyzed by Raman imaging, with a capability to detect single NPs below 100 nm (*9*). In terms of plastic particle emitted from road dust, no comparable result was reported from the saltation process during wind erosion of surface soils. Input of a sharp increase in plastic amounts into models may lead to reassessments and insights into processes that transfer plastics between the atmosphere and marine or terrestrial environments (*15*, *16*, *22*). Detailed chemical and microphysical characterization of individual MP and NP particles may facilitate a complete balance for global plastics, advance the parameterization of atmospheric processes, and better identify the negative impacts of plastic accumulation.

### Atmospheric NP particles

NP particles in the atmosphere are believed to size-dependently influence global climate (*15*), atmospheric chemistry (*17*), toxicity (*22*), and the regional and global transport of pollutants and biological nutrients (*46*) in a distinct way from MPs (*5*, *8*). The atmospheric NPs are unintentionally produced in the environment and are thus physicochemically distinguishable from manufactured nanomaterials (*8*). Because of a wide variety of sources, NPs are polydisperse in physical properties and heterogeneous in chemical composition (*47*). Results from tracking and quantifying NPs must be interpreted cautiously, particularly when methodologies advanced for nanomaterials are adopted to study environmental NPs.

Within atmospheric aerosols, nanoparticles are typically formed via gas-to-particle conversion and act as potential precursors for particle growth, often dominating the total particle number concentration (*48*). Although the detection limit of CCSEM is 200 nm, our results unexpectedly revealed low number concentrations of atmospheric NPs in both megacities, with abundances and fluxes one order of magnitude lower than those of MPs in most environmental samples (Fig. 1). While aerosol collection efficiency decreased with particle size (reaching a minimum of 73.5% for particles in the 300- to 350-nm range), the impinger captured a substantially higher number of submicrometer particles (table S2). Nevertheless, the abundance peak in the size distribution of plastic particles consistently occurred within the micrometer-size range across all atmospheric samples (Fig. 2). A conventional understanding attributed the origin of MPs and NPs primarily to the progressive fragmentation of larger plastics resulting from physical (e.g., mechanical wear, heat, and ultraviolet), chemical (e.g., pH and ions), and biological degradation (*5*, *8*). These pathways are potentially interconnected: photooxidation, thermooxidation, and biooxidation weaken polymer structures and facilitate fragmentation. Continued weathering modifies debris surface morphology and active sites, thereby increasing chemical reactivity.

Although our CCSEM system detects particles as small as 200 nm, no NPs below 260 nm were observed in any environmental samples. The average NP size in the urban atmosphere ranged from 649 ± 174 nm in dustfall to 809 ± 121 nm in rainwater, both recorded in XA (Table 1). All samples showed average NP sizes between 600 and 800 nm. These findings challenge earlier assumptions that plastic particles gradually fragment into tens of nanometers under low mineralization rates. Instead, environmental processes such as degradation and mineralization convert plastic carbon into nonpolymeric forms, including gases and soluble organic matter (*14*). Ultraviolet-induced radical oxidation reduces polymer molecular weight and enhances water solubility through carboxyl and hydroxyl group formation (*49*). In addition, photodegradation-released dissolved organic carbon is rapidly consumed by bacteria (*50*). Although laboratory studies have detected NPs as small as tens of nanometers (*5*, *7*–*9*, *32*, *47*, *51*), which is below our detection limit, the absence of 200- to 260-nm particles in real atmospheric environments suggests a potential decomposition process of ultrafine fragments into soluble or mineralized compounds.

### Heteroaggregation of atmospheric plastic particles

**Fig. 3.**
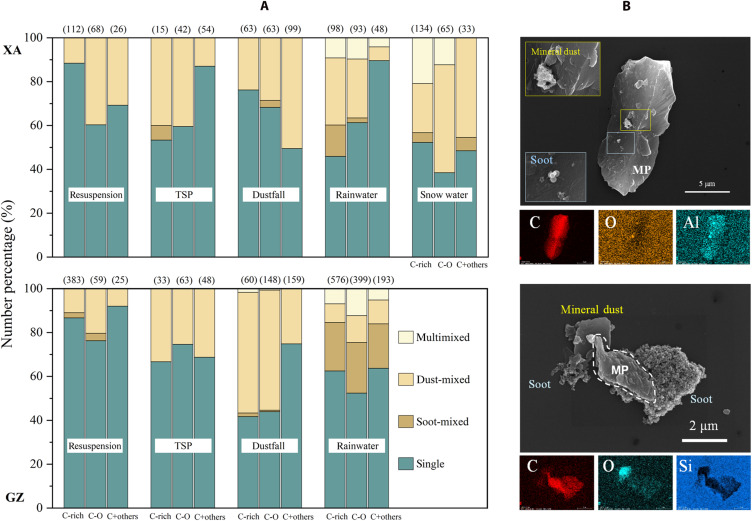
Plastic aggregate mixing states across atmospheric compartments in XA and GZ city. (**A**) Normalized abundance of plastics aggregated with mineral dust and/or soot (black carbon). (**B**) Representative SEM micrographs and EDX elemental mappings of plastic aggregates with micro- and nanoscale aerosols. Top: Snow-sourced plastic aggregate from XA showing nanoscale attachments. Bottom: Rainwater-sourced plastic aggregate from XA with microscale interlocked components.

Upon release into or formation within the atmosphere, both MPs and NPs are highly prone to heteroaggregation with airborne aerosol particles originating from natural sources (e.g., mineral dust) or anthropogenic pollution (e.g., soot) (*8*, *17*). Although our pretreatment procedures removed biological and water-soluble materials, current evidence confirms the presence of plastic aggregates in all environmental samples. These aggregates exhibit preferential heteroaggregation with specific types of airborne particulates across a wide range of particle sizes. EDX mappings revealed plastic heteroaggregates with soot and/or mineral dusts, which occurred at a frequency of up to 65.7% (Fig. 3). Mineral dust-plastic aggregates occurred ubiquitously and were most abundant in TSP and deposition samples. While soot represents a dominant urban air pollutant, soot-plastic aggregates primarily occurred in wet deposition samples. Multicomponent aggregates (plastic simultaneously aggregated with both soot and mineral dust) were enriched specifically in rainwater and snow, likely due to water-mediated hydrogen bonding during in-cloud heteroaggregation. Critically, all samples underwent aqueous pretreatment, confirming that observed variations in aggregate composition and abundance reflect authentic atmospheric processes rather than methodological artifacts.

Multiple mechanisms likely govern mutual sorption between particulate plastics and aerosols in urban atmospheres, involving anthropogenic colloids (e.g., soot) and natural geogenic materials (e.g., mineral dust) (*52*). Heteroaggregation of environmental plastics, acting as either sorbates or sorbents, primarily facilitates the interfacial interactions between particle surfaces. These interactions include electrostatic forces, Van der Waals forces, hydrogen bonding, π-π interactions, and formation of chemical bonding via functional group associations (*8*, *17*). Given the frequent observation of heterogeneous surface speciation within individual aggregates (Fig. 3), single heteroaggregation systems typically involve concurrent physical and chemical mechanisms.

Mineral dust is ubiquitous in the atmosphere, providing reactive substrates for heterogeneous reactions or medium on and in which reactions can occur (*53*). Hydrophobic and hydrophilic soot particles coexist as effective vectors for mineral dust, organic matter, and water vapor while serving as polyfunctional catalytic substrates (*54*–*56*). Plastic materials, inherently diverse in polymer/additive composition, are chemically inert, physically durable, and predominantly hydrophobic (*17*, *22*). Upon heteroaggregation with soot and/or mineral dust in atmospheric environments, both plastic particles and their aerosol counterparts undergo substantial alterations in physicochemical properties. Heteroaggregation alters plastic particles’ size (Fig. 2), shape (figs. S3 to S5), and density (fig. S6) while inducing heterogeneous chemical modifications to surface active sites (Fig. 3). These transformations critically determine their radiative forcing, transport pathways, and chemical reactivity across atmospheric, marine, and terrestrial systems.

### Plastic emission from urban road dust

Even during stable, low-wind conditions, megacity traffic remains a potentially major source of atmospheric MPs and NPs through the continuous resuspension of road dust. An integrated analysis combining in situ observations, atmospheric transport modeling, and optimal estimation techniques revealed that resuspended road dust dominates MP sources in the western United States, accounting for 84% of terrestrial MP deposition (*13*). In addition, MPs from road traffic exhibit high long-range transport efficiency to remote regions (*57*). Traffic-induced turbulence and wind-driven road dust saltation emit substantial quantities of primarily plastic particles, most of which remain single in both micrometer and submicrometer scales. In both studied cities, only chamber-resuspended road dust contained relatively high NP abundances and emission fluxes (Fig. 1), indicating wind erosion of surface soil as an important NP production mechanism. Analogous to fine mineral dust generation through wind erosion in arid regions (*58*), NP enrichment occurs via fragmentation of heteroaggregated plastics in road dust through saltation and sandblasting processes. Furthermore, urban resuspensions showed substantially fewer plastic aggregates than other atmospheric samples (Fig. 3 and fig. S7), confirming that heteroaggregation occurs predominantly after particle emission into the air. Observed abundance variations across emission, aerosol, and deposition phases likely reflect the following: (i) recirculating air mass transport patterns, (ii) aging transformations, and (iii) aggregation processes within strong vertical flux regimes.

### Limitations and future work

Dozens of research papers are published each year, investigating the effects of environmental exposure to MPs and NPs on microbial cycles, chemical transformations, ecosystems, and human health. Yet a critical prerequisite remains unquantified: the size-resolved abundance of atmospheric plastic particles and their fluxes across Earth’s environmental compartments. Major efforts are currently underway to develop rapid and reliable methods for identifying, quantifying, and characterizing atmospheric MPs and NPs in environmental samples. This challenge is compounded by a continuous spectrum of particle properties, including size, shape, density, and chemical composition, as well as their strong tendency to heteroaggregate with both natural and anthropogenic particles.

While this study provides a semiautomated quantification method for MPs and NPs across multiple urban environmental media, several limitations should be acknowledged. First, the EDX-based approach has inherent limitations in distinguishing polymer types. Second, the reliance on interpretable EDX signals restricted the detectable size of plastic particles to those larger than 200 nm (table S1). Third, the pretreatment procedures designed to exclude nonplastic carbonaceous particles also resulted in the removal of biological materials and water-soluble organic and inorganic substances present in the environmental samples. Consequently, the absolute number concentration, flux, and heteroaggregating states of plastic particles are more reliable than relative abundance values, especially given that samples were not collected simultaneously. At the same time, although our approach yields a direct measure of total wet deposition flux, it precludes the separation of in-cloud versus below-cloud scavenging, which is essential for predicting atmospheric particle lifetimes. Last, the current semiautomated method remains time-consuming, requiring extended periods (tens of hours per sample) for particle relocation and manual differentiation of plastics from soot aggregates. As a result, TSP samples collected over five consecutive days were pooled into composite samples, and measurements from other media were based on only a single sample. This precluded any assessment of standard deviation across individual environmental samples.

Despite these limitations, this study offers a quantitative assessment of atmospheric plastics within complex environmental matrices, the least understood reservoir in the global plastic cycle. Future efforts should prioritize the development of faster and more accurate analytical methods. Bulk analytical techniques, such as mass spectrometry, are unable to measure particle morphology or perform repeated analyses, as the particles are destroyed during the volatilization of their components. In contrast, microscopy-based methods preserve the structural integrity of plastic particles, enabling complementary analyses that are essential for polymer speciation and quantification. As complementary techniques, Raman spectroscopy coupled with SEM allows for single-particle polymer identification (*33*, *51*). However, current approaches rely on random visual inspection and lack robust quantitative capability. By evaluating the advantages and limitations of both CCSEM and Raman techniques, we propose that integrating a Raman detector with CCSEM could effectively overcome these challenges. This approach will enable absolute quantification of individual plastic particles through the following: (i) SEM-based characterization of size and morphology, (ii) EDX-guided particle relocation, and (iii) Raman confirmation of chemical identity.

## MATERIALS AND METHODS

### Experimental design

At the elemental level, plastics are predominantly carbon. However, the applicability of SEM-EDX as a standalone technique for quantifying plastic particles is fundamentally limited (*59*). This limitation arises because environmental samples contain a vast array of carbon-containing particles, both organic (e.g., biological debris, and soot) and inorganic (e.g., carbonate minerals), that SEM-EDX cannot distinguish from plastic based solely on elemental composition. Herein, we applied a series of inclusion and exclusion criteria on the basis of the assumption that atmospheric carbon-containing particles are dominated by biological materials (tissues), carbonate minerals, tar balls, soot aggregates (black carbon), carbonaceous fly ash, and plastic particles. Accordingly, a combination of digestion procedures (to remove organisms, tar balls, and carbonates) and morphological screening (to differentiate soot aggregates and carbonaceous fly ash) was adopted to separate plastic from nonplastic carbon-containing particles (fig. S8). In the digestion pretreatment, tar balls were readily removed because of their water solubility (*60*, *61*), biological tissues were digested with hydrogen peroxide (*26*, *62*), and subsequent acid digestion ensured dissolution of carbonate minerals. Subsequent morphology-based identification used a CCSEM system to relocate individual carbon-containing particles. Soot aggregates were identified by their chain-like or clustered morphology with primary particle diameters of 10 to 500 nm (*63*, *64*). Spheroidal carbonaceous fly ash was recognized on the basis of spongy or porous structures (*65*–*67*). At this stage, remaining carbon-containing particles were classified as plastic and manually double checked one by one to confirm carbon composition, surface textures, and morphologies. The CCSEM dataset, containing elemental and morphological data from thousands of individual particles per sample, enables statistical quantification of atmospheric MPs/NPs’ chemical and microphysical properties.

It should be noted that a key constraint of the EDX-based CCSEM technique is its inability to determine chemical bonding. Thus, quantification of atmospheric plastics is subject to uncertainties resulting from challenges in distinguishing carbon-based plastics from primary organic aerosol (POA) and secondary organic aerosol (SOA) particles. Uncertainties from POAs’ interferences are likely negligible, as most POAs are primary engineered nanomaterials (<100 nm) (*8*, *52*) that fall below the detection limit of this method. The primary interference arises from SOAs. SOAs originate either from the condensation of anthropogenic waste emissions (*68*, *69*) or from the oxidation of volatile organic compounds and semivolatile organic compounds in the atmosphere (*70*). These SOA particles, either aggregates in the atmosphere or secondary organic coatings on preexisting aerosols, exhibit diverse size distributions that overlap with plastic particles. EDX analyses indicate that SOA particles are composed mainly of carbon, with smaller contributions from sulfur, chloride, and other inorganic elements (*71*), and closely mimic that of plastics in the C + others subgroup. Consequently, despite the fact that aqueous pretreatment may remove some water-soluble organics and the high-voltage electron beam and high-vacuum environment during microanalysis can cause the loss of volatile species, the remaining SOA components are likely to introduce unquantifiable uncertainties into the quantification of atmospheric plastics in environmental samples, particularly for particles >200 nm within the C + others subgroup.

### Environmental samples

Aerosol, dry and wet deposition, and road dust samples were collected in two megacities in China (fig. S9). One site was in XA, the most populous city in northwestern China, with a population of nearly 13.17 million in 2024. The other site was in GZ, the largest city in South China, with a population of nearly 18.98 million in 2024. Both sampling sites were located near the main ring road of the respective city with heavy traffic, namely Xingqing Road in XA (34.24°N, 108.98°E) and Hengfu Road in GZ (23.13°N, 113.28°E).

Aerosol particles (TSPs) were collected using a liquid impinger method (fig. S10), with double-distilled water (DDW) as the collection medium. Particles were captured with an all-glass midget impinger (SKC, 50 ml, standard nozzle) containing 20 ml of DDW. An air sampling pump (SKC, Universal Pump 44XR) maintained a constant airflow rate of 1 liter min^−1^ for 60 min. Impinger samples collected over five consecutive days in each city (XA: 18 to 22 March 2024; GZ: 13 to 17 January 2024) were merged in a 500-ml glass conical flask. Impinger walls were triple-rinsed with DDW (10 ml for each rinse). Procedural blanks were collected concurrently with the pump deactivated. Aerosol collection efficiency for particles of 0.3 to 10 μm in aerodynamic diameter (table S2) was quantified using an optical particle sizer (TSI Model 3330) connected via a drying tube to the exhaust port of the SKC pump. Relative humidity at the optical particle sizer inlet was maintained at 20 ± 5%, monitored downstream of the drying tube using an indoor air quality monitor (Q-Trak, TSI Model 7575).

Road dust was swept using soft-bristle brushes into self-made aluminum foil shovels and transferred to aluminum foil bags. Resuspended road dust aerosols were generated using a laboratory dust simulation system designed to replicate natural wind erosion saltation processes from surface soils (*58*). Approximately 100 g of road dust was evenly spread on an aluminum alloy sheet within the chamber. Resuspension was initiated at a blade rotation rate of 1200 rpm, producing an equivalent friction velocity of 0.54 m s^−1^ for dust mobilization (*57*). Total resuspended particulate matter was collected using the liquid impinger method described previously (fig. S11).

Dry and wet deposition samples were collected in glass bulk deposition collectors (length: 20 cm; width: 10 cm; height: 10 cm) positioned 1.5 m above ground level. Dustfall sampling occurred from December 2023 to January 2024. After collection, particles were transferred to 200-ml conical flasks through triple rinsing with 10 ml of DDW per rinse. Wet deposition sampling commenced 0.5 hours after precipitation initiation and continued for exactly 1 hour. Rainwater and snowmelt volumes (XA only) were measured using a graduated pipette before transfer to 200-ml conical flasks, with triple rinsing of beakers using 10 ml of DDW. All samples were immediately covered with aluminum foil and refrigerated at 4°C until processing.

All samples used in this study were collected from public sidewalks in XA and GZ city. These locations are public areas open to everyone, and the collection of such samples does not require any specific permit or approval. The sampling processes did not involve any protected species or restricted ecological environments.

### Sample pretreatment

Before microanalysis, all samples underwent a three-step pretreatment procedure on conductive silicon wafers (10 by 10 by 0.5 mm, Santechem, Taizhou, China) to remove interferents (fig. S12): (i) In water dialysis for salt/tar ball removal, aqueous suspensions were handshaken for 1 min, and sample aliquots were transferred via glass micropipettes (Blaubrand intraMARK, Brand, Wertheim, Germany) onto silicon wafers. Residual salt/tar ball removal was enhanced through extended wafer immersion in dialyzed water (*72*). Wafers were then dried for 48 hours at room temperature in vacuum desiccators. (ii) In peroxide digestion of biological material, 10 ml of aqueous hydrogen peroxide (30% H_2_O_2_, Sinopharm Chemical Reagent Co. Ltd., Shanghai, China) was added into the petri dish. Being immersed in the solution for 24 hours, the silicon wafer was vacuum dried. (iii) In acid digestion of carbonates, wafers were exposed to dropwise-added 1% HCl (Xilong Scientific Co., Ltd., Chengdu, China; ~10 drops min^−1^ until they were immersed). After pretreatment, the silicon wafer was mounted onto a SEM aluminum specimen stub (fig. S13) for microanalysis.

### Semiautomated microanalysis

On the basis of EDX analyses of more than 6000 individual particles within each environmental sample, our CCSEM system identified and located all carbon-containing particles among them. An automated positioning procedure was subsequently performed, thereby ensuring that all targets were relocatable for morphological verification. Particles with the distinct morphologies of soot aggregates or carbonaceous fly ash were manually excluded, leading to the classification of the remaining carbon-containing particles as plastics.

The automated microanalysis system comprises a field-emission gun scanning electron microscope (MAIA3, TESCAN, Brno, Czech Republic) equipped with an EDX spectrometer (XFlash 6 I 60, BRUKER, Karlsruhe, Germany) and CCSEM software (IntelliSEM Environment Particle Analysis System, RJ Lee Group Inc., Pittsburgh, US). SEM was performed in high-vacuum mode with working distances between 5 and 10 mm under an accelerating voltage of 20 kV. To obtain particle images with a clear outline and repeatable EDX spectra, only particles with their diameter larger than 200 nm were measured without coating. The elemental compositions of a particle were presented as weight percentages of detected elements, including C, O, F, Na, Mg, Al, P, S, Cl, K, Ca, Ti, Mn, Fe, Cu, Zn, and Br, which were normalized on the basis of their EDX characteristic x-ray spectra.

Particle diameters were measured using a rotated-feret box technique, with a box or caliper being rotated in a clockwise manner around the particles and the particle length being recorded at each orientation. The diameter of a particle (*D*_p_) is the arithmetic average of all the 90 measurements, and the maximum diameter (*d*_max_) is the largest of all the measurements. The aspect ratio of a particle is the ratio of the longest dimension (*d*_max_) of the particle to its longest perpendicular width (*d*_perp_). The roundness (*R*) of a particle indicates its trend to resemble a circle with an area equivalent to the projected area (*A*) of the particle. Roundness was calculated using the following formula

$$R=\frac{4A}{\pi d_{\text{max}}^{2}}$$ (1)

The form factor (FF) of a particle is a dimensionless number relating to its area and outline perimeter (*p*), indicating the roughness of the particle outline, and is given by

$$\text{FF}=\frac{4\pi A}{p^{2}}$$ (2)

Aerosol particle number concentrations were calculated from the analyzed areas (SEM view fields), number of particles analyzed, total volume of impinged liquid to the particle flow for TSP samples or resuspended road dust samples, sampling duration, and flow regime. Particle number concentrations of dry and wet deposition were calculated from the CCSEM-analyzed areas, total volume of rainwater or melt snow water, time exposure, and the horizontal area of passive samplers.

Carbon contamination arises from the deposition of carbon during EDX analysis as a result of thermal cracking of hydrocarbons at and near the electron beam impact point (*73*). Given our use of conductive silicon wafers (nominal Si content >99.90 wt %) as CCSEM substrates, this contamination presents a substantial concern for plastic detection under EDX conditions. These conditions require high-resolution analysis of trace transition metals used as polymer additives. Given that measured carbon contamination levels on silicon wafers remained below 6.0 wt % (table S3), we used a conservative carbon content threshold of 10.0 wt % for positive plastic identification in this method.

By manually excluding all nonplastic carbon-containing particles, two distinct particle categories remained in each environmental sample (table S4). Mineral dust particles with carbon content <10.0 wt % in their EDX spectra were categorized into the “C-excluded” group. “Plastic” particles were defined by a carbon content reaching 10.0 wt % in their EDX spectra. Plastic particles were further classified into three subgroups on the basis of elemental composition: (i) C-rich plastics (C ≥ 99.5 wt %) represent polymers like PE, PP, PS, and rubber. (ii) C + O plastics (C + O ≥ 99.5 wt %) represent oxygen-containing polymers, primarily PET, PC, PVA, PMMA, and epoxy resin. (iii) C + others plastics encompass plastics containing notable elements beyond C and O. This includes polymers constituted by monomers containing F, Cl, or N (e.g., PVC, PTFE, PU, and PA), as well as plastics containing additives like antioxidants, plasticizers, flame retardants, surfactants, or other elements introduced during manufacturing.

Method precision was validated through triplicate measurements of laboratory-synthesized C_3_N_4_ particles on both identical and separate regions of a silicon wafer, which yielded reproducible particle size distributions and detection rates (table S5). The particle sizing accuracy of the CCSEM system was further confirmed by measuring more than 6000 monodisperse polystyrene latex reference spheres.

### Estimation of plastic fluxes

Using the MP and NP particle counts from each sample aliquot, we estimated the atmospheric plastic abundances and their fluxes across environmental media as follows. The number concentrations of MPs and NPs in TSPs are given by

$$c_{\text{aerosol}}=\frac{N_{\text{aerosol}}}{V_{\text{aerosol}}}\times \frac{\left(V_{\text{DDW}}+V_{\text{rinse}}\right)}{\left(\nu \times t\right)}$$ (3)

where *c*_aerosol_ is the number concentration of MP or NP in TSP samples (unit: MPs m^−3^ or NPs m^−3^), *V*_aerosol_ is the total volume (500 μl) of an aliquot of five TSP samples dropped onto silicon wafers, *N*_aerosol_ is the MP or NP particle count, *V*_DDW_ is the total volume (100 ml) of DDW in the impinger, *V*_rinse_ is the total volume (150 ml) of DDW for impinger rinsing, *t* is the total sampling duration (300 min) in five consecutive sampling days, and ν is the air flow rate (1 liter min^−1^) of the sampler.

The emission fluxes of MPs and NPs via road dust resuspensions are given by

$$F_{\text{resus}}=\frac{N_{\text{resus}}}{V_{\text{resus}}}\times \frac{\left(V_{\text{DDW}}+V_{\text{rinse}}\right)}{\left(\nu \times t\right)}\times \frac{Q}{A_{\text{chamber}}}$$ (4)

where *F*_resus_ is the emission flux of MP or NP from road dust resuspensions (unit: MPs m^−2^ day^−1^ or NPs m^−2^ day^−1^), *N*_resus_ is the MP or NP particle count, *V*_resus_ is the volume (500 μl) of resuspension sample aliquot dropped onto silicon wafers, *V*_DDW_ is the volume (20 ml) of DDW in the impinger, *V*_rinse_ is the volume (30 ml) of DDW for impinger rinsing, *t* is the sampling duration (46.7 min) for resuspension samples, ν is the air flow rate (1 liter min^−1^) of the sampler, *Q* is the air flow rate (250 liters min^−1^) exhausted from the resuspension chamber, and *A*_chamber_ is the bottom area (0.255 m^2^) of the suspension chamber.

The dry deposition fluxes of MPs and NPs are given by

$$F_{\text{dry}}=\frac{N_{\text{dry}}}{V_{\text{dry}}}\times \frac{V_{\text{DDW}}}{\left(A_{\text{collector}}\times t\right)}$$ (5)

where *F*_dry_ is the dry deposition flux of MP or NP (unit: MPs m^−2^ day^−1^ or NPs m^−2^ day^−1^), *N*_dry_ is the MP or NP particle count, *V*_dry_ is the volume (100 μl) of the dustfall suspension aliquot dropped onto silicon wafers, *V*_DDW_ is the volume (30 ml) of DDW for collector rinsing, *A*_collector_ is the bottom area (0.02 m^2^) of the glass bulk deposition collector, and *t* is the sampling duration (234 hours in XA and 169 hours in GZ) for dry deposition collections.

The wet deposition fluxes of MPs and NPs are as the following formula, in which the term for *A*_collector_ (bottom area of the glass bulk deposition collector) cancels out

$$F_{\text{wet}}=\frac{N_{\text{wet}}}{V_{\text{wet}}}\times P$$ (6)

where *F*_wet_ is the wet deposition flux of MP or NP (unit: MPs m^−2^ day^−1^ or NPs m^−2^ day^−1^), *N*_wet_ is the MP or NP particle count, *V*_wet_ is the volume (100 μl) of rainwater or snow water (XA only) sample aliquot dropped onto silicon wafers, *P* is the precipitation rate (0.000267 mm hour^−1^ during the snowfall between 23:00 and 24:00 on 11 November 2023 in XA, averaged 1.45 mm hour^−1^ during the rainfall between 20:00 and 21:00 on 10 December 2023 in XA, and averaged 0.46 mm hour^−1^ during the rainfall between 22:00 and 23:00 on 22 January 2024 in GZ) during each 1-hour sampling period.

### Quality assurance and quality control

Avoiding impurity introduction is critical for robust quality control and assurance in plastic detection. Throughout environmental sample collection, processing, and analysis, all plastics were strictly excluded. Carbon-containing materials were avoided, including filters (e.g., polycarbonate filter, cellulose acetate microfiber filter, and polytetrafluoroethylene filter), glove (made of nitrile, latex, neoprene, or vinyl), and plastic apparatuses, containers, gasket seals, and tools. All sample-contacting items were replaced with glass, aluminum foil, or stainless-steel alternatives and thoroughly rinsed twice with DDW before use. Airborne contamination was minimized by covering samples with aluminum foil, conducting work within laminar flow cabinets, and wearing natural fiber clothing (100% cotton).

Exposure of plastic particles in suspensions or solutions to ultrasonic oscillation is known to induce three key alterations, including reduction in particle size (*74*), chemical degradation (*75*), and dissipation of agglomerates and aggregates (*76*). Furthermore, mechanical fragmentation is recognized as a major source of environmental MPs and NPs (*3*, *16*). We observed fragmentation of plastic reference materials subjected to both ultrasonic baths and electromagnetic stirring. These treatments yielded broken particles and smaller debris exhibiting diverse sizes and shapes (fig. S14). Consequently, only manual handshaking was performed for sample mixing in this protocol.

Given the ubiquity of plastic particles and their potential for sample contamination, rigorous quality assurance procedures, including blanks, spike recovery, and contamination prevention, are critical for reliable environmental plastic particle analysis (*59*, *77*–*79*). Cross-contamination from laboratory materials and solutions during sample processing represents a major contamination source. To address this, we quantified background plastic levels in analytical solvents, reagents, and spiked samples. Procedural blanks were subsequently measured for each environmental sample to quantitatively represent procedural contamination levels in this method.

### Contamination of analytical solvents and reagents

Distilled water and deionized water (DIW) are common analytical solvents in MP sample preparation. Distillation removes impurities with boiling points higher than water, while deionization passes source water through oppositely charged ion-exchange resins. Here, DDW (laboratory DDW) and DIW (Milli-Q water; resistivity, 18.0 MΩ cm^−1^) were purified through quartz filters, dropped onto silicon wafer via graduated pipettes, and analyzed by CCSEM-EDX. DIW contained higher particulate carbon concentrations [(1.4 ± 0.7) × 10^6^ particles liter^−1^; size range: 0.4 to 74.2 μm] compared to DDW [(1.3 ± 0.4) × 10^6^ particles liter^−1^; size range: 0.6 to 47.2 μm]. These notable abundances exceed values from manual μ-Raman/micro–Fourier transform infrared spectrometry analyses (*77*, *80*). Carbon-containing particles in DIW were predominantly plastics, likely originating from fragmentation of ion-exchange resins (fig. S15A). Plastics in DDW primarily derived from debris in bottles/tubes (fig. S15B). All analytical solvents underwent triplicate microanalysis, with glass containers triple-rinsed using 0.5 liters of DDW per rinse.

Similar to analytical solvents, laboratory reagents are often produced in plastic-uncontrolled environments and packaged in plastic containers, introducing severe plastic contamination into analyses (*77*, *81*, *82*). To quantify this, we diluted hydrogen peroxide (30% H_2_O_2_) and hydrochloric acid (36 to 38% HCl) with DDW and performed microanalysis (fig. S16). Results revealed MP concentrations of (5.0 ± 1.7) × 10^4^ particles liter^−1^ in hydrogen peroxide and (1.3 ± 0.3) × 10^5^ particles liter^−1^ in hydrochloric acid.

### Spike recovery

Spiked samples were applied to validate the effectiveness of our sample preparation and automated microanalysis. Recovery rates varied across spike types because of differences in morphological consistency and chemical stability. We used laboratory reference materials (soot and polystyrene latex) and commercial polymer micropowders (PTFE, PVC, PA6, PP, PE, and PET) with particle sizes ranging from hundreds of nanometers to micrometers. Table S6 details all particulate-carbon reference materials and their recovery rates for both digestion pretreatment and microanalysis.

All reference materials retained their original morphologies (fig. S17A) following water dialysis (fig. S17B) and H_2_O_2_ digestion (fig. S17C). However, HCl digestion induced notable alterations in PE, including particle size reduction and the emergence of nanoscale surface grains (fig. S17D). Consequently, for samples containing minimal mineral carbonate particles (identified by EDX spectra showing colocalized C, O, Ca, and/or Mg), the HCl digestion step was omitted to prevent chemical degradation of susceptible plastics. Mineral carbonates in these samples were instead manually identified via their diagnostic EDX spectral signatures.

### Procedural blanks

Uncertainty in MP and NP identification and quantification arises from contamination during field collection, laboratory preparation, and analysis. Therefore, procedural blanks were used to assess and correct for procedural contamination after the comparative assessment of particle abundances in analytical solvents, reagents, and spiked samples. For each environmental medium, including aerosols, dry/wet deposition, and resuspended road dust, a total of nine procedural blanks from GZ and XA were obtained (table S7). The mean plastic particle counts per blank type were subtracted from corresponding city-specific analytical results.

## References

[1] R. C. Thompson, W. Courtene-Jones, J. Boucher, S. Pahl, K. Raubenheimer, A. A. Koelmans, Twenty years of microplastics pollution research—What have we learned? Science 386, eadl2746 (2024). 39298564 10.1126/science.adl2746

[2] A. L. Dawson, S. Kawaguchi, C. K. King, K. A. Townsend, R. King, W. M. Huston, S. M. Bengtson Nash, Turning microplastics into nanoplastics through digestive fragmentation by Antarctic krill. Nat. Commun. 9, 1001 (2018). 29520086 10.1038/s41467-018-03465-9 PMC5843626

[3] K. Zhang, A. H. Hamidian, A. Tubić, Y. Zhang, J. K. Fang, C. Wu, P. K. Lam, Understanding plastic degradation and microplastic formation in the environment: A review. Environ. Pollut. 274, 116554 (2021). 33529891 10.1016/j.envpol.2021.116554

[4] R. C. Thompson, Y. Olsen, R. P. Mitchell, A. Davis, S. J. Rowland, A. W. G. John, D. McGonigle, A. E. Russell, Lost at sea: Where is all the plastic? Science 304, 838–838 (2004). 15131299 10.1126/science.1094559

[5] J. Gigault, A. T. Halle, M. Baudrimont, P.-Y. Pascal, F. Gauffre, T.-L. Phi, H. El Hadri, B. Grassl, S. Reynaud, Current opinion: What is a nanoplastic? Environ. Pollut. 235, 1030–1034 (2018). 29370948 10.1016/j.envpol.2018.01.024

[6] N. B. Hartmann, T. Hüffer, R. C. Thompson, M. Hassellöv, A. Verschoor, A. E. Daugaard, S. Rist, T. Karlsson, N. Brennholt, M. Cole, M. P. Herrling, M. Heß, N. P. Lvleva, A. L. Lusher, M. Wagner, Are we speaking the same language? Recommendations for a definition and categorization framework for plastic debris. Environ. Sci. Technol. 53, 1039–1047 (2019). 30608663 10.1021/acs.est.8b05297

[7] N. P. Ivleva, Chemical analysis of microplastics and nanoplastics: Challenges, advanced methods, and perspectives. Chem. Rev. 121, 11886–11936 (2021). 34436873 10.1021/acs.chemrev.1c00178

[8] J. Gigault, H. El Hadri, B. Nguyen, B. Grassl, L. Rowenczyk, N. Tufenkji, S. Feng, M. Wiesner, Nanoplastics are neither microplastics nor engineered nanoparticles. Nat. Nanotechnol. 16, 501–507 (2021). 33927364 10.1038/s41565-021-00886-4

[9] N. Qian, X. Gao, X. Lang, H. Deng, T. M. Bratu, Q. Chen, P. Staoleton, B. Yan, W. Min, Rapid single-particle chemical imaging of nanoplastics by SRS microscopy. Proc. Natl. Acad. Sci. U.S.A. 121, e2300582121 (2024). 38190543 10.1073/pnas.2300582121 PMC10801917

[10] M. Bergmann, S. Mützel, S. Primpke, M. B. Tekman, J. Trachsel, G. Gerdts, White and wonderful? Microplastics prevail in snow from the Alps to the Arctic. Sci. Adv. 5, eaax1157 (2019). 31453336 10.1126/sciadv.aax1157 PMC6693909

[11] L. Yang, W. Luo, P. Zhao, Y. Zhang, S. Kang, J. P. Giesy, F. Zhang, Microplastics in the Koshi River, a remote alpine river crossing the Himalayas from China to Nepal. Environ. Pollut. 290, 118121 (2021). 34523512 10.1016/j.envpol.2021.118121

[12] I. Peeken, S. Primpke, B. Beyer, J. Gütermann, C. Katlein, T. Krumpen, M. Bergmann, L. Hehemann, G. Gerdts, Arctic sea ice is an important temporal sink and means of transport for microplastic. Nat. Commun. 9, 1505 (2018). 29692405 10.1038/s41467-018-03825-5 PMC5915590

[13] J. Brahney, N. Mahowald, M. Prank, G. Cornwell, Z. Klimont, H. Matsui, K. A. Prather, Constraining the atmospheric limb of the plastic cycle. Proc. Natl. Acad. Sci. U.S.A. 118, e2020719118 (2021). 33846251 10.1073/pnas.2020719118 PMC8072239

[14] A. Stubbins, K. L. Law, S. E. Muñoz, T. S. Bianchi, L. Zhu, Plastics in the Earth system. Science 373, 51–55 (2021). 34210876 10.1126/science.abb0354

[15] L. E. Revell, P. Kuma, E. C. Le Ru, W. R. C. Somerville, S. Gaw, Direct radiative effects of airborne microplastics. Nature 598, 462–467 (2021). 34671134 10.1038/s41586-021-03864-x

[16] D. Allen, S. Allen, S. Abbasi, A. Baker, M. Bergmann, J. Brahney, T. Butler, R. A. Duce, S. Eckhardt, N. Evangeliou, T. Jickells, M. Kanakidou, P. Kershaw, P. Laj, J. Levermore, D. Li, P. Liss, K. Liu, N. Mahowald, P. Masque, D. Materić, A. G. Mayes, P. McGinnity, I. Osvath, K. A. Prather, J. M. Prospero, L. E. Revell, S. G. Sander, W. J. Shim, J. Slade, A. Stein, O. Tarasova, S. Wright, Microplastics and nanoplastics in the marine-atmosphere environment. Nat. Rev. Earth Environ. 3, 393–405 (2022).

[17] M. Aeschlimann, G. Li, Z. A. Kanji, D. M. Mitrano, Potential impacts of atmospheric microplastics and nanoplastics on cloud formation processes. Nat. Geosci. 15, 967–975 (2022). 36532143 10.1038/s41561-022-01051-9 PMC7613933

[18] Y. Zhang, T. Gao, S. Kang, H. Shi, L. Mai, D. Allen, S. Allen, Current status and future perspectives of microplastic pollution in typical cryospheric regions. Earth Sci. Rev. 226, 103924 (2022).

[19] P. Brahana, M. Zhang, E. Nakouzi, B. Bharti, Weathering influences the ice nucleation activity of microplastics. Nat. Commun. 15, 9579 (2024). 39505887 10.1038/s41467-024-53987-8 PMC11542094

[20] T. M. Seifried, S. Nikkho, A. M. Murillo, L. J. Andrew, E. R. Grant, A. K. Bertram, Microplastic particles contain ice nucleation sites that can be inhibited by atmospheric aging. Environ. Sci. Technol. 58, 15711–15721 (2024). 39172764 10.1021/acs.est.4c02639 PMC11375776

[21] S. L. Wright, F. J. Kelly, Plastic and human health: A micro issue? Environ. Sci. Technol. 51, 6634–6647 (2017). 28531345 10.1021/acs.est.7b00423

[22] A. A. Koelmans, P. E. Redondo-Hasselerharm, N. H. M. Nor, V. N. de Ruijter, S. M. Mintenig, M. Kooi, Risk assessment of microplastic particles. Nat. Rev. Mater. 7, 138–152 (2022).

[23] J. C. Prata, J. P. da Costa, I. Lopes, A. C. Duarte, T. Rocha-Santos, Environmental exposure to microplastics: An overview on possible human health effects. Sci. Total Environ. 702, 134455 (2020). 31733547 10.1016/j.scitotenv.2019.134455

[24] S. Abbasi, B. Keshavarzi, F. Moore, A. Turner, F. J. Kelly, A. O. Dominguez, N. Jaafarzadeh, Distribution and potential health impacts of microplastics and microrubbers in air and street dusts from Asaluyeh County, Iran. Environ. Pollut. 244, 153–164 (2019). 30326387 10.1016/j.envpol.2018.10.039

[25] S. Wieland, A. Balmes, J. Bender, J. Kitzinger, F. Meyer, A. F. Ramsperger, F. Roeder, C. Tengelmann, B. H. Wimmer, C. Laforsch, H. Kress, From properties to toxicity: Comparing microplastics to other airborne microparticles. J. Hazard. Mater. 428, 128151 (2022). 35042167 10.1016/j.jhazmat.2021.128151

[26] B. Nguyen, D. Claveau-Mallet, L. M. Hernandez, E. G. Xu, J. M. Farner, N. Tufenkji, Separation and analysis of microplastics and nanoplastics in complex environmental samples. Acc. Chem. Res. 52, 858–866 (2019). 30925038 10.1021/acs.accounts.8b00602

[27] Y. Zhang, S. Kang, S. Allen, D. Allen, T. Gao, M. Sillanpää, Atmospheric microplastics: A review on current status and perspectives. Earth Sci. Rev. 203, 103118 (2020).

[28] X. Y. Sheng, Y. J. Lai, S. J. Yu, Q. C. Li, Q. X. Zhou, J. F. Liu, Quantitation of atmospheric suspended polystyrene nanoplastics by active sampling prior to pyrolysis–gas chromatography–mass spectrometry. Environ. Sci. Technol. 57, 10754–10762 (2023). 37428629 10.1021/acs.est.3c02299

[29] G. Chen, Z. Fu, H. Yang, J. Wang, An overview of analytical methods for detecting microplastics in the atmosphere. TrAC Trends Anal. Chem. 130, 115981 (2020).

[30] S. Primpke, S. H. Christiansen, W. Cowger, H. De Frond, A. Deshpande, M. Fischer, E. B. Holland, M. Meyns, B. A. O’Donnell, B. E. Ossmann, M. Pittroff, G. Sarau, B. M. Scholz-Böttcher, K. J. Wiggin, Critical assessment of analytical methods for the harmonized and cost-efficient analysis of microplastics. Appl. Spectrosc. 74, 1012–1047 (2020). 32249594 10.1177/0003702820921465

[31] E. Caracci, A. Vega-Herrera, J. Dachs, N. Berrojalbiz, G. Buonanno, E. Abad, M. Llorca, T. Moreno, M. Farré, Micro (nano) plastics in the atmosphere of the Atlantic Ocean. J. Hazard. Mater. 450, 131036 (2023). 36857820 10.1016/j.jhazmat.2023.131036

[32] Z. Sobhani, X. Zhang, C. Gibson, R. Naidu, M. Megharaj, C. Fang, Identification and visualisation of microplastics/nanoplastics by Raman imaging (i): Down to 100 nm. Water Res. 174, 115658 (2020). 32146170 10.1016/j.watres.2020.115658

[33] G. Sarau, L. Kling, B. E. Oßmann, A. K. Unger, F. Vogler, S. H. Christiansen, Correlative microscopy and spectroscopy workflow for microplastics. Appl. Spectrosc. 74, 1155–1160 (2020). 32186214 10.1177/0003702820916250

[34] G. Xu, H. Cheng, R. Jones, Y. Feng, K. Gong, K. Li, X. Fang, M. A. Tahir, V. K. Valev, L. Zhang, Surface-enhanced Raman spectroscopy facilitates the detection of microplastics < 1 μm in the environment. Environ. Sci. Technol. 54, 15594–15603 (2020). 33095569 10.1021/acs.est.0c02317

[35] L. Chang, S. Jiang, J. Luo, J. Zhang, X. Liu, C. Y. Lee, W. Zhang, Nanowell-enhanced Raman spectroscopy enables the visualization and quantification of nanoplastics in the environment. Environ. Sci. Nano 9, 542–553 (2022).

[36] Y. Su, X. Hu, H. Tang, K. Lu, H. Li, S. Liu, B. Xing, R. Ji, Steam disinfection releases micro (nano) plastics from silicone-rubber baby teats as examined by optical photothermal infrared microspectroscopy. Nat. Nanotechnol. 17, 76–85 (2022). 34764453 10.1038/s41565-021-00998-x

[37] L. Xie, S. Luo, Y. Liu, X. Ruan, K. Gong, Q. Ge, K. Li, V. K. Valev, G. Liu, L. Zhang, Automatic identification of individual nanoplastics by Raman spectroscopy based on machine learning. Environ. Sci. Technol. 57, 18203–18214 (2023). 37399235 10.1021/acs.est.3c03210

[38] H. Cai, E. G. Xu, F. Du, R. Li, J. Liu, H. Shi, Analysis of environmental nanoplastics: Progress and challenges. Chem. Eng. J. 410, 128208 (2021).

[39] L. Xie, K. Gong, Y. Liu, L. Zhang, Strategies and challenges of identifying nanoplastics in environment by surface-enhanced raman spectroscopy. Environ. Sci. Technol. 57, 25–43 (2023). 36576086 10.1021/acs.est.2c07416

[40] G. S. Casuccio, “Particle population analysis by automated scanning electron microscopy” in *Microanalysis of Atmospheric Particles: Techniques and Applications* (John Wiley & Sons, 2025), pp. 91–112.

[41] Y. V. Paramitadevi, A. Turyanti, E. Rishanti, B. Ratnawati, B. S. Ramadan, N. Ikhlas, “Atmospheric microplastic transport” in *Microplastics in the Ecosphere: Air, Water, Soil, and Food* (Wiley, 2023), pp. 77–95.

[42] C. M. Rochman, T. Hoellein, The global odyssey of plastic pollution. Science 368, 1184–1185 (2020). 32527817 10.1126/science.abc4428

[43] M. MacLeod, H. P. H. Arp, M. B. Tekman, A. Jahnke, The global threat from plastic pollution. Science 373, 61–65 (2021). 34210878 10.1126/science.abg5433

[44] W. Huang, X. Xia, Element cycling with micro (nano) plastics. Science 385, 933–935 (2024). 39208108 10.1126/science.adk9505

[45] X. Zhu, W. Huang, M. Fang, Z. Liao, Y. Wang, L. Xu, Q. Mu, C. Shi, C. Lu, H. Deng, R. Dahlgren, X. Shang, Airborne microplastic concentrations in five megacities of northern and southeast China. Environ. Sci. Technol. 55, 12871–12881 (2021). 34559513 10.1021/acs.est.1c03618

[46] J. E. Bullard, A. Ockelford, P. O’Brien, C. M. Neuman, Preferential transport of microplastics by wind. Atmos. Environ. 245, 118038 (2021).

[47] S. Wagner, T. Reemtsma, Things we know and don’t know about nanoplastic in the environment. Nat. Nanotechnol. 14, 300–301 (2019). 30944425 10.1038/s41565-019-0424-z

[48] C. Anastasio, S. T. Martin, Atmospheric nanoparticles. Rev. Mineral. Geochem. 44, 293–349 (2001).

[49] M. P. Born, C. Brüll, From model to nature—A review on the transferability of marine (micro-) plastic fragmentation studies. Sci. Total Environ. 811, 151389 (2022). 34808157 10.1016/j.scitotenv.2021.151389

[50] L. Zhu, S. Zhao, T. B. Bittar, A. Stubbins, D. Li, Photochemical dissolution of buoyant microplastics to dissolved organic carbon: Rates and microbial impacts. J. Hazard. Mater. 383, 121065 (2020). 31518809 10.1016/j.jhazmat.2019.121065

[51] W. Zhang, Z. Dong, L. Zhu, Y. Hou, Y. Qiu, Direct observation of the release of nanoplastics from commercially recycled plastics with correlative Raman imaging and scanning electron microscopy. ACS Nano 14, 7920–7926 (2020). 32441911 10.1021/acsnano.0c02878

[52] T. Hüffer, A. Praetorius, S. Wagner, F. Von der Kammer, T. Hofmann, Microplastic exposure assessment in aquatic environments: Learning from similarities and differences to engineered nanoparticles. Environ. Sci. Technol. 51, 2499–2507 (2017). 28125881 10.1021/acs.est.6b04054

[53] C. R. Usher, A. E. Michel, V. H. Grassian, Reactions on mineral dust. Chem. Rev. 103, 4883–4940 (2003). 14664636 10.1021/cr020657y

[54] T. S. G. A. B. Novakov, S. G. Chang, A. B. Harker, Sulfates as pollution particulates: Catalytic formation on carbon (soot) particles. Science 186, 259–261 (1974). 17782021 10.1126/science.186.4160.259

[55] B. Zuberi, K. S. Johnson, G. K. Aleks, L. T. Molina, M. J. Molina, A. Laskin, Hydrophilic properties of aged soot. Geophys. Res. Lett. 32, L01807 (2005).

[56] K. A. Koehler, P. J. DeMott, S. M. Kreidenweis, O. B. Popovicheva, M. D. Petters, C. M. Carrico, E. D. Kireeva, T. D. Khokhlova, N. K. Shonija, Cloud condensation nuclei and ice nucleation activity of hydrophobic and hydrophilic soot particles. Phys. Chem. Chem. Phys. 11, 7906–7920 (2009). 19727498 10.1039/b905334b

[57] V. Etyemezian, G. Nikolich, S. Ahonen, M. Pitchford, M. Sweeney, R. Purcell, J. Gillies, H. Kuhns, The portable in situ wind erosion laboratory (PI-SWERL): A new method to measure PM10 windblown dust properties and potential for emissions. Atmos. Environ. 41, 3789–3796 (2007).

[58] F. Wu, Y. Cheng, T. Hu, N. Song, F. Zhang, Z. Shi, S. S. H. Ho, J. Cao, D. Zhang, Saltation–sandblasting processes driving enrichment of water-soluble salts in mineral dust. Environ. Sci. Technol. Lett. 9, 921–928 (2022).

[59] B. Oßmann, D. Schymanski, N. P. Ivleva, D. Fischer, F. Fischer, G. Dallmann, F. Welle, Comment on “exposure to microplastics (< 10 μm) associated to plastic bottles mineral water consumption: The first quantitative study by Zuccarello et al. [Water Research 157 (2019) 365–371]”. Water Res. 162, 516–517 (2019). 31255329 10.1016/j.watres.2019.06.032

[60] J. L. Hand, W. C. Malm, A. Laskin, D. Day, T. B. Lee, C. Wang, C. Carrico, J. Carrillo, J. P. Cowin, J. Collett Jr., M. J. Iedema, Optical, physical, and chemical properties of tar balls observed during the Yosemite Aerosol characterization study. J. Geophys. Res. Atmos. 110, D21210 (2005).

[61] A. V. Tivanski, R. J. Hopkins, T. Tyliszczak, M. K. Gilles, Oxygenated interface on biomass burn tar balls determined by single particle scanning transmission X-ray microscopy. Chem. A Eur. J. 111, 5448–5458 (2007). 10.1021/jp070155u 17542565

[62] M. T. Nuelle, J. H. Dekiff, D. Remy, E. Fries, A new analytical approach for monitoring microplastics in marine sediments. Environ. Pollut. 184, 161–169 (2014). 24051349 10.1016/j.envpol.2013.07.027

[63] J. Lahaye, G. Prado, “Morphology and internal structure of soot and carbon blacks” in *Particulate Carbon: Formation During Combustion* (Springer New York, 1981), pp. 33–55.

[64] J. Davis, K. Tiwari, I. Novosselov, Soot morphology and nanostructure in complex flame flow patterns via secondary particle surface growth. Fuel 245, 447–457 (2019). 31736504 10.1016/j.fuel.2019.02.058 PMC6858054

[65] M. Wik, I. Renberg, Environmental records of carbonaceous fly-ash particles from fossil-fuel combustion. J. Paleolimnol. 15, 193–206 (1996).

[66] P. Ausset, M. Del Monte, R. A. Lefevre, Embryonic sulphated black crusts on carbonate rocks in atmospheric simulation chamber and in the field: Role of carbonaceous fly-ash. Atmos. Environ. 33, 1525–1534 (1999).

[67] N. L. Rose, Spheroidal carbonaceous fly ash particles provide a globally synchronous stratigraphic marker for the Anthropocene. Environ. Sci. Technol. 49, 4155–4162 (2015). 25790111 10.1021/acs.est.5b00543

[68] A. C. Morales, J. M. Tomlin, C. P. West, F. A. Rivera-Adorno, B. N. Peterson, S. A. Sharpe, Y. Noh, S. M. T. Sendesi, B. Boor, J. A. Howarter, R. C. Moffet, S. China, B. T. O’Callahan, P. Z. El-Khoury, A. J. Whelton, A. Laskin, Atmospheric emission of nanoplastics from sewer pipe repairs. Nat. Nanotechnol. 17, 1171–1177 (2022). 36203091 10.1038/s41565-022-01219-9

[69] H. Shen, X. Wang, A. K. Lee, J. P. Abbatt, A. W. Chan, Nanoplastic particle emissions from plastic smoldering combustion. Environ. Sci. Technol. 59, 19339–19350 (2025). 40899375 10.1021/acs.est.5c05852

[70] R. Zhang, G. Wang, S. Guo, M. L. Zamora, Q. Ying, Y. Lin, W. Wang, M. Hu, Y. Wang, Formation of urban fine particulate matter. Chem. Rev. 115, 3803–3855 (2015). 25942499 10.1021/acs.chemrev.5b00067

[71] V. Berta, L. M. Russell, “Microscopy methods for organic composition of atmospheric aerosol particles” in *Microanalysis of Atmospheric Particles: Techniques and Applications* (American Geophysical Union, 2025), pp. 177–199.

[72] D. Zhang, Y. Iwasaka, Size change of Asian dust particles caused by sea salt interaction: Measurements in southwestern Japan. Geophys. Res. Lett. 31, L15102 (2004).

[73] B. Buse, S. Kearns, Importance of carbon contamination in high-resolution (FEG) EPMA of silicate minerals. Microsc. Microanal. 21, 594–605 (2015). 25877617 10.1017/S1431927615000288

[74] J. R. Peller, S. P. Mezyk, S. Shidler, J. Castleman, S. Kaiser, R. F. Faulkner, C. D. Pilgrim, A. Wilson, S. Martens, G. P. Horne, Facile nanoplastics formation from macro and microplastics in aqueous media. Environ. Pollut. 313, 120171 (2022). 36113647 10.1016/j.envpol.2022.120171

[75] B. Miljevic, F. Hedayat, S. Stevanovic, K. E. Fairfull-Smith, S. E. Bottle, Z. D. Ristovski, To sonicate or not to sonicate PM filters: Reactive oxygen species generation upon ultrasonic irradiation. Aerosol Sci. Tech. 48, 1276–1284 (2014).

[76] S. Kefer, T. Friedenauer, H. C. Langowski, Characterisation of different manufactured plastic microparticles and their comparison to environmental microplastics. Powder Technol. 412, 117960 (2022).

[77] J. C. Prata, V. Reis, J. P. da Costa, C. Mouneyrac, A. C. Duarte, T. Rocha-Santos, Contamination issues as a challenge in quality control and quality assurance in microplastics analytics. J. Hazard. Mater. 403, 123660 (2021). 33264868 10.1016/j.jhazmat.2020.123660

[78] V. C. Shruti, G. Kutralam-Muniasamy, Blanks and bias in microplastic research: Implications for future quality assurance. Trends Environ. Anal. Chem. 38, e00203 (2023).

[79] Y. Liu, J. Han, Y. Wang, A. Li, J. Zhao, Y. Su, L. Shen, B. Xing, Suspected sources of microplastics and nanoplastics: Contamination from experimental reagents and solvents. Water Res. 249, 120925 (2024). 38039819 10.1016/j.watres.2023.120925

[80] J. Yang, M. Monnot, Y. Sun, L. Asia, P. Wong-Wah-Chung, P. Doumenq, P. Moulin, Microplastics in different water samples (seawater, freshwater, and wastewater): Methodology approach for characterization using micro-FTIR spectroscopy. Water Res. 232, 119711 (2023). 36796150 10.1016/j.watres.2023.119711

[81] C. Way, M. D. Hudson, I. D. Williams, G. J. Langley, Evidence of underestimation in microplastic research: A meta-analysis of recovery rate studies. Sci. Total Environ. 805, 150227 (2022). 34537704 10.1016/j.scitotenv.2021.150227

[82] R. Bai, R. Fan, C. Xie, Q. Liu, C. Yan, J. Cui, W. He, Microplastics are overestimated due to poor quality control of reagents. J. Hazard. Mater. 459, 132068 (2023). 37494798 10.1016/j.jhazmat.2023.132068

