# Supplementary material for: Abundance of microplastics and nanoplastics in urban atmosphere
Source: Sci Adv. 2026 Jan 7;12(2):eadz7779. doi: 10.1126/sciadv.adz7779 (PMC12778049; doi:10.1126/sciadv.adz7779)
Supplement: Supplementary file 1 — Figs. S1 to S17 Tables S1 to S7 [file sciadv.adz7779_sm.pdf]

Science Advances
AAAS

Supplementary Materials for

Abundance of microplastics and nanoplastics in urban atmosphere

Tafeng Hu *et al.*

Corresponding author: Tafeng Hu, hutf@ieecas.cn; Yu Huang, huangyu@ieecas.cn;
Kin-Fai Ho, kfho@cuhk.edu.hk; Junji Cao, jjcao@mail.iap.ac.cn; Daizhou Zhang, dzzhang@pu-kumamoto.ac.jp

Sci. Adv. **12**, eadz7779 (2026)
DOI: 10.1126/sciadv.adz7779

This PDF file includes:

Figs. S1 to S17
Tables S1 to S7

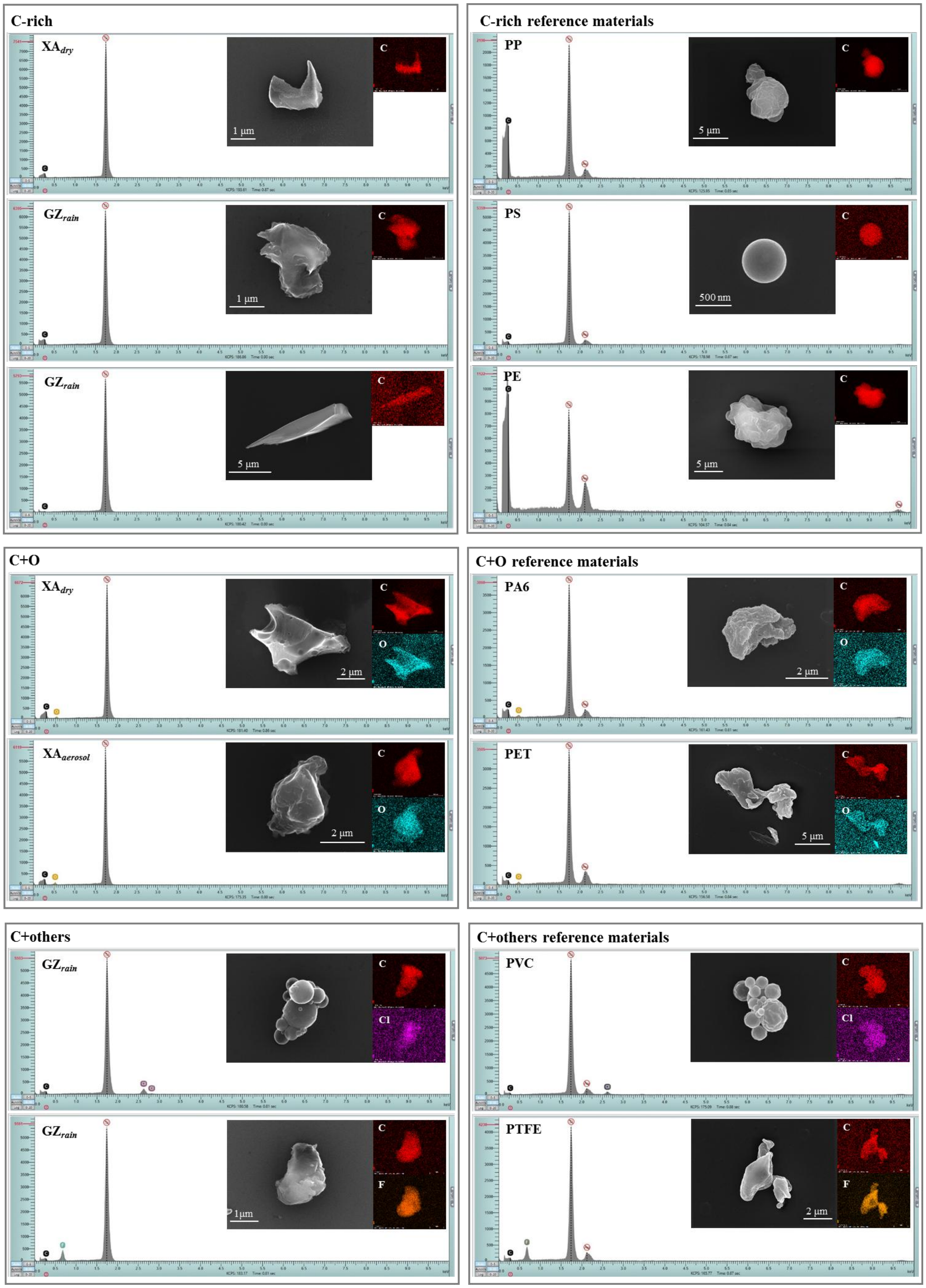

Fig. S1. EDX spectra and elemental mappings of representative MPs from environmental samples and their corresponding reference materials for each polymer subgroup. The Si signal originates from the silicon wafer substrate, while the Au signal comes from the gold coating applied for electrical conductivity.

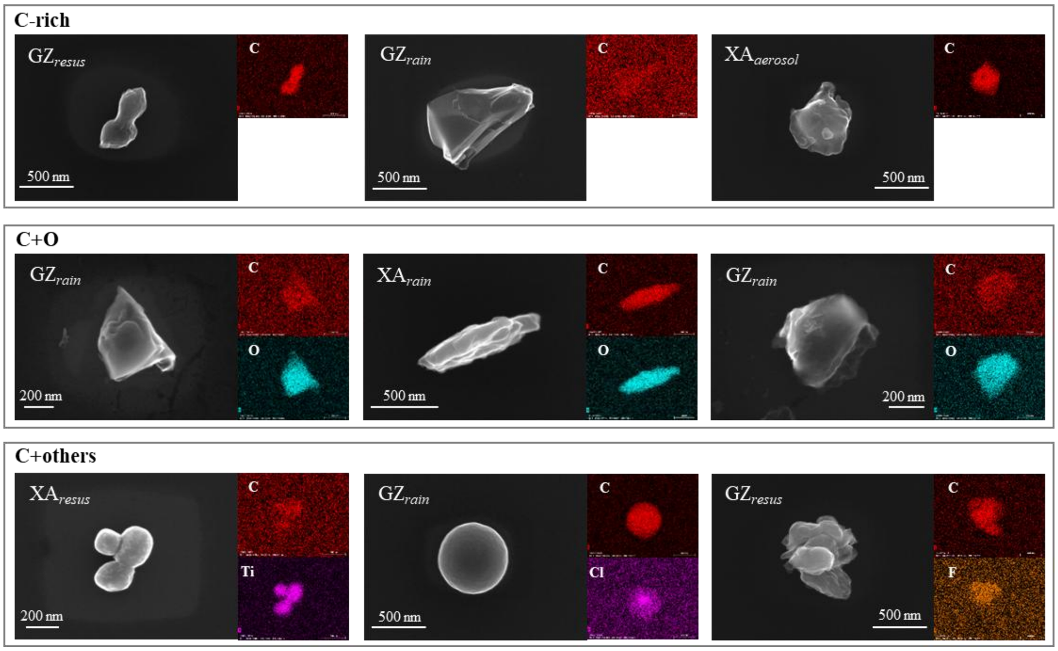

Fig. S2. Representative morphologies and EDX elemental mappings of NPs in each polymer subgroup.

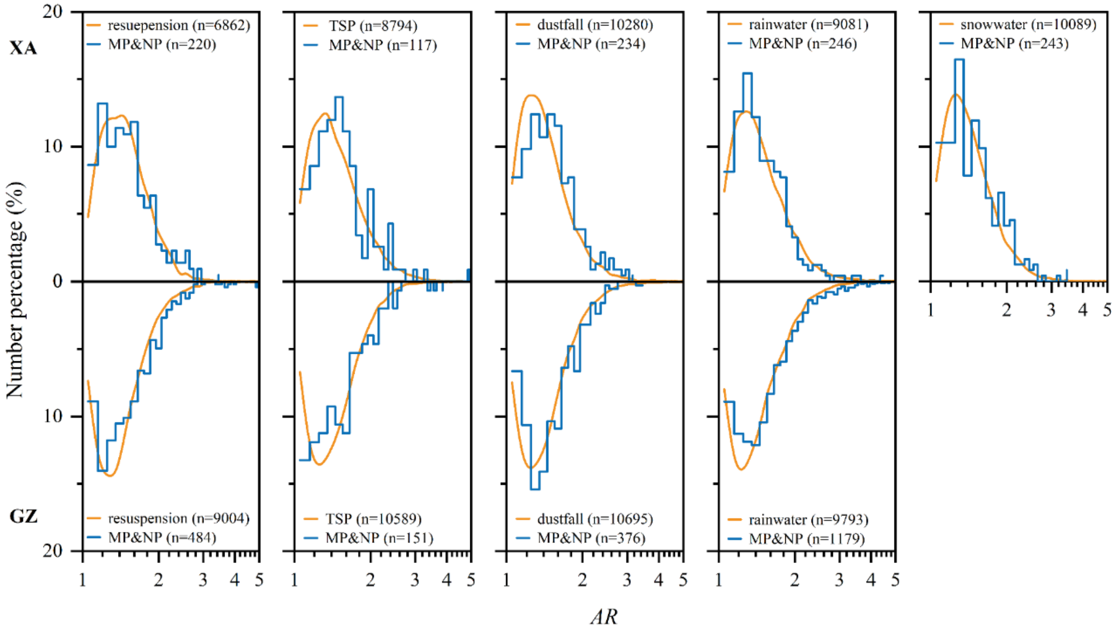

Fig. S3. Particle aspect ratio (*AR*) distribution of atmospheric MPs and NPs across urban compartments in Xi'an and Guangzhou. Particle abundance stratified by *AR* group. The numerical values in parentheses for each sample correspond to the total particle counts (orange curves) and the plastic-only counts (blue lines). The total particle count encompasses both plastic and non-plastic particles.

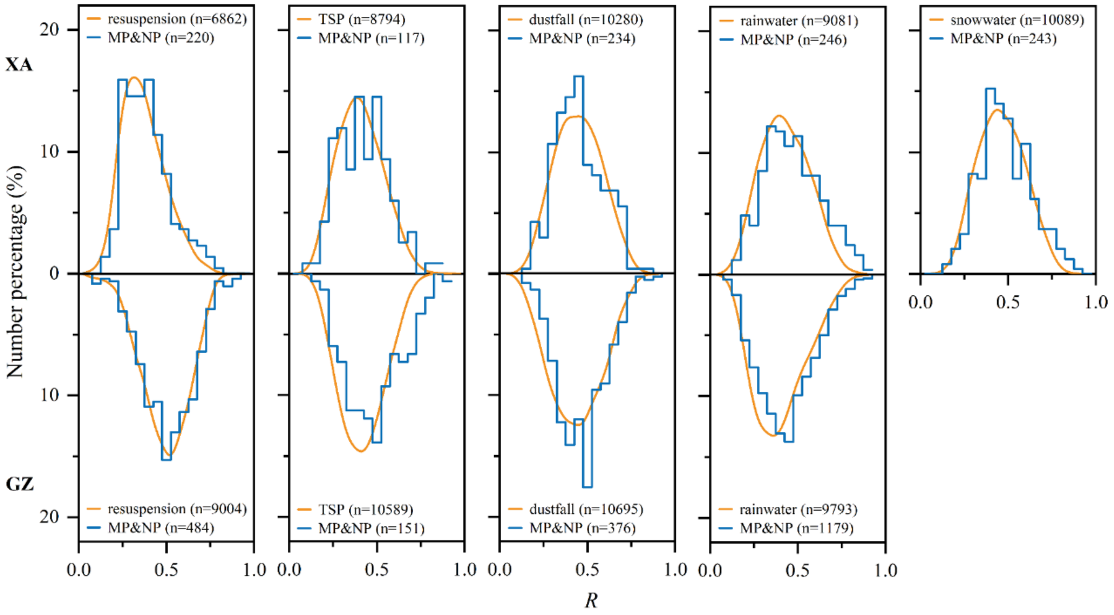

Fig. S4. Particle roundness (*R*) distribution of atmospheric MPs and NPs across urban compartments in Xi'an and Guangzhou. Particle abundance stratified by *R* group. The numerical values in parentheses for each sample correspond to the total particle counts (orange curves) and the plastic-only counts (blue lines). The total particle count encompasses both plastic and non-plastic particles.

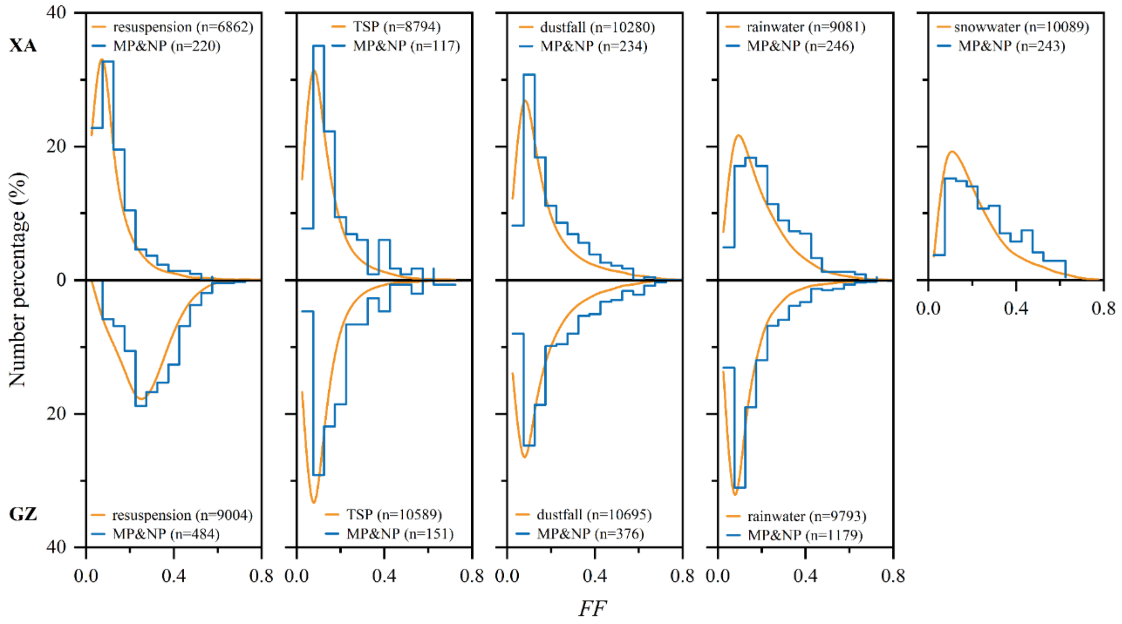

Fig. S5. Particle form factor (*FF*) distribution of atmospheric MPs and NPs across urban compartments in Xi'an and Guangzhou. Particle abundance stratified by *FF* group. The numerical values in parentheses for each sample correspond to the total particle counts (orange curves) and the plastic-only counts (blue lines). The total particle count encompasses both plastic and non-plastic particles.

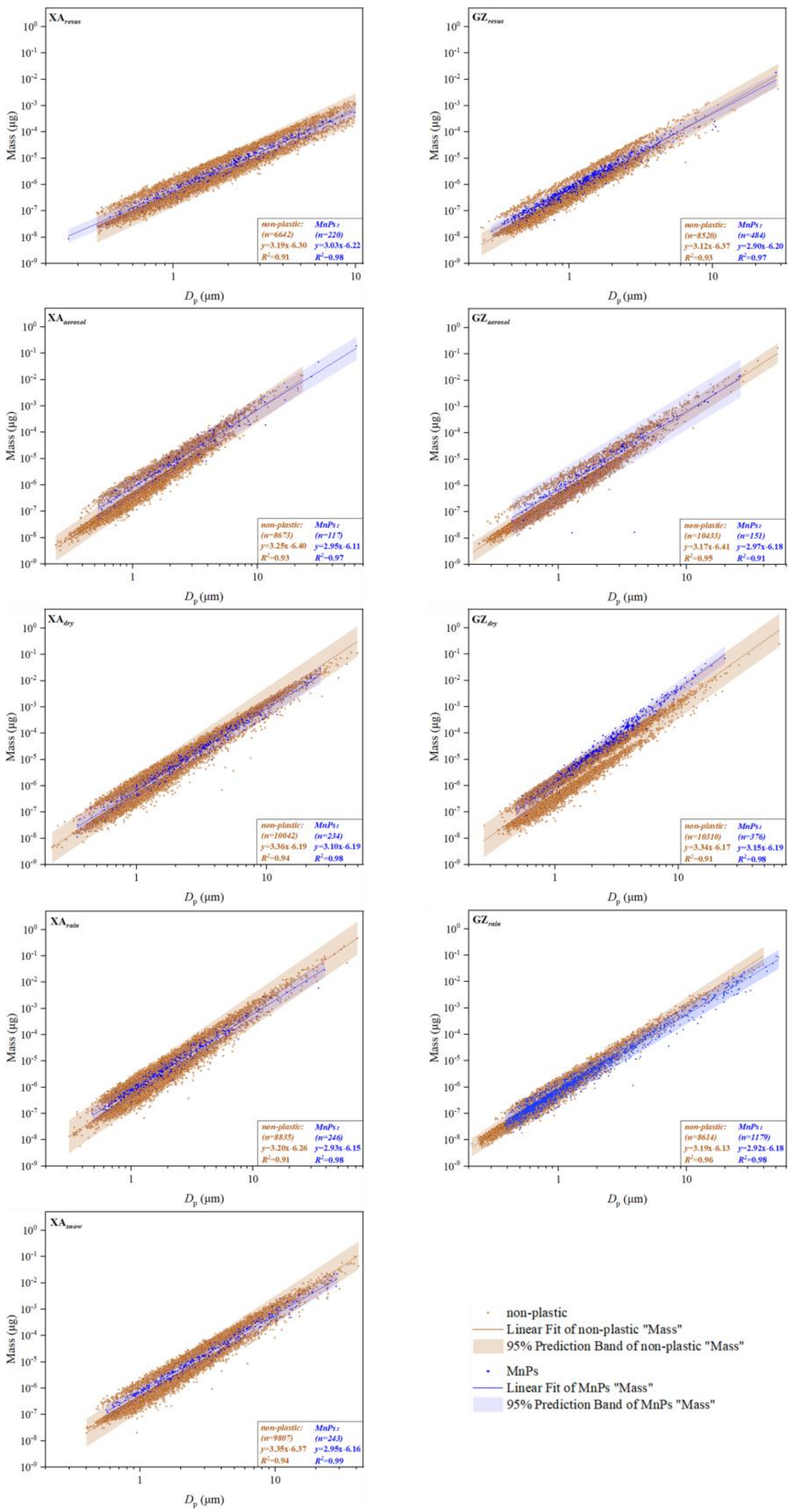

Fig. S6. Individual particle mass as a function of particle size. The mass of each particle was derived on the basis of a series of assumptions pertaining to equivalent spherical volume and density as a function of elemental composition.

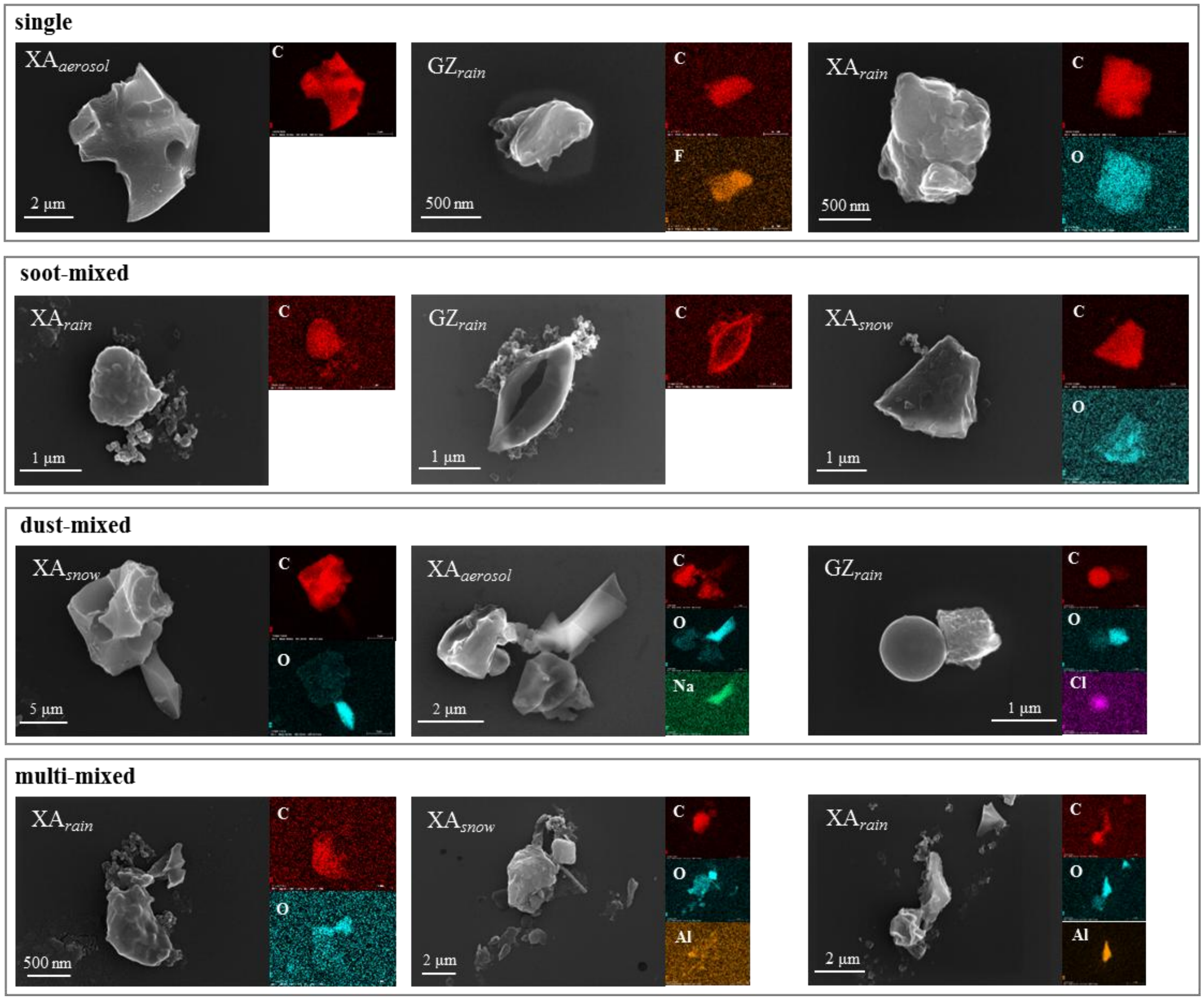

Fig. S7. Representative micrographs and EDX elemental mappings of plastic aggregates.

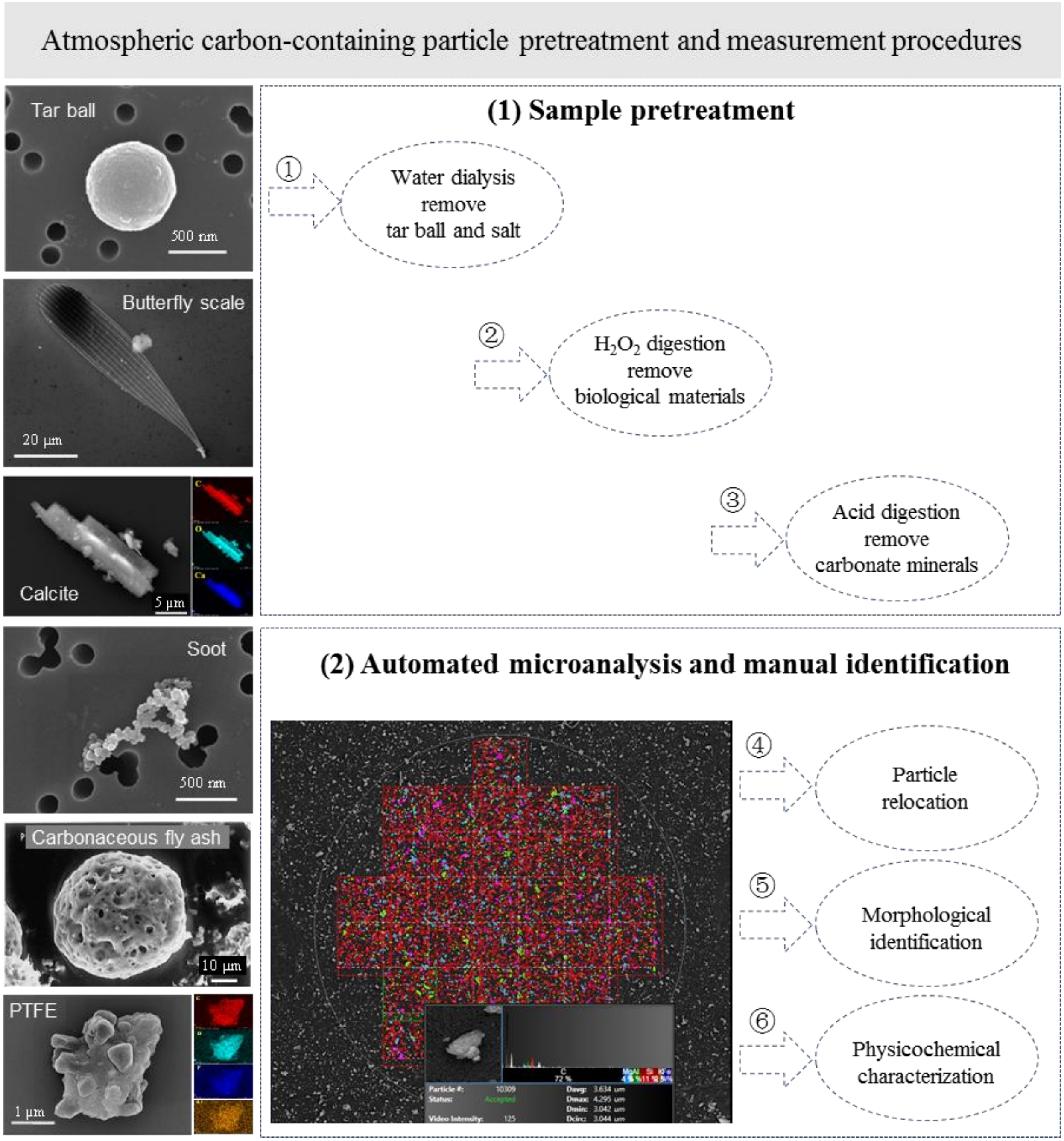

Fig. S8. Experimental design schematic. The representative carbonaceous fly ash image is sourced from Reference 66 [Reprinted from Atmospheric Environment, Vol 33, P. Ausset, M. Del Monte, R. A. Lefevre, Embryonic sulphated black crusts on carbonate rocks in atmospheric simulation chamber and in the field: role of carbonaceous fly-ash, 1525-1534, Copyright (1999), with permission from Elsevier]. All other components of this figure were created by the authors. PTFE, polytetrafluoroethene.

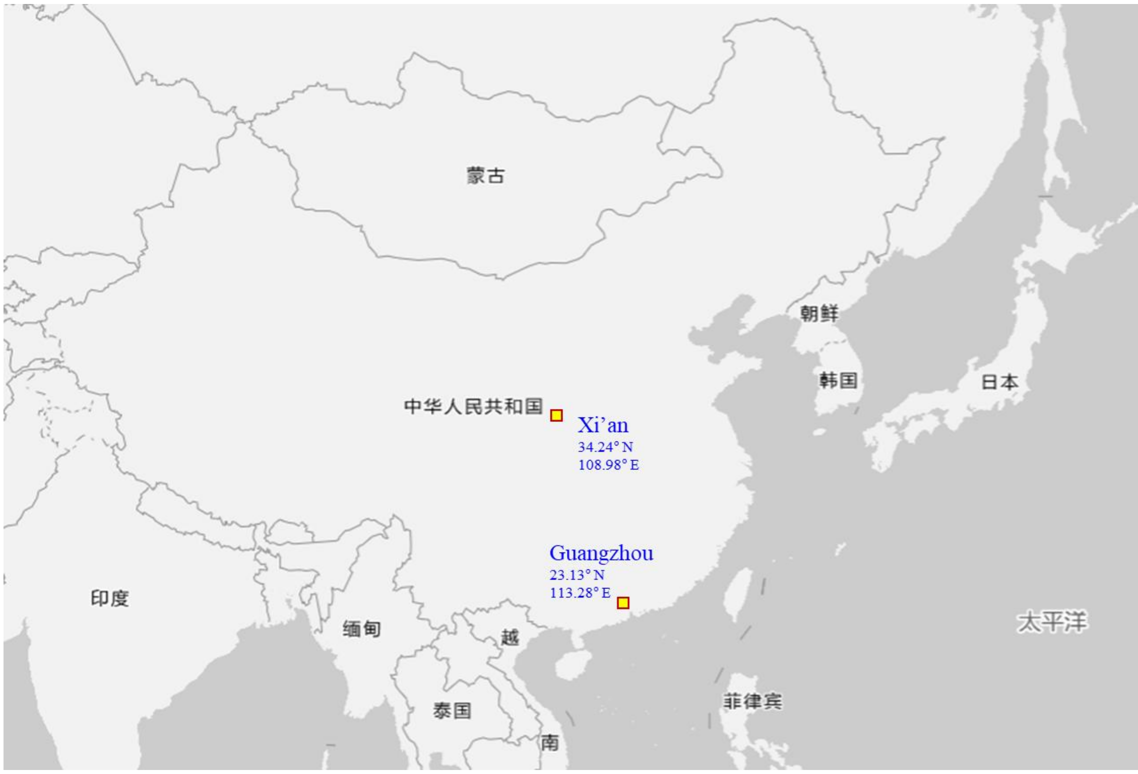

Fig. S9. Locations of our sampling sites in Xi’an and Guangzhou. Map source: https://map.tianditu.gov.cn/ [map approval number: GS (2025) 1508]

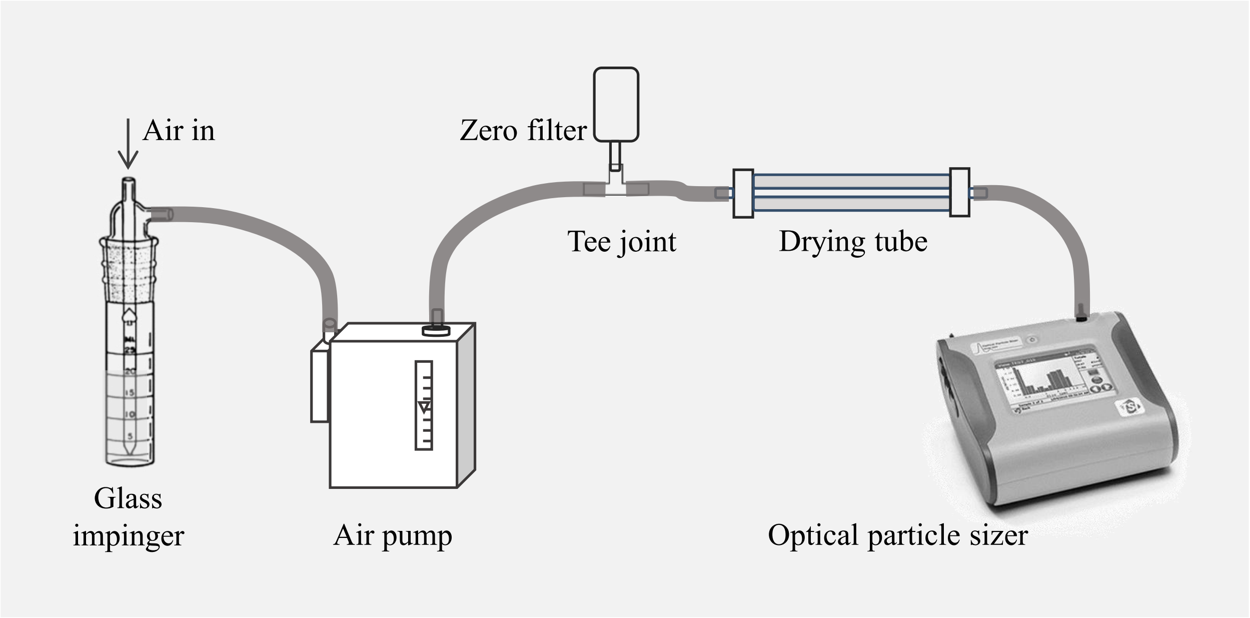

Fig. S10. Schematic of aerosol sampling and the evaluation of aerosol collection efficiency.

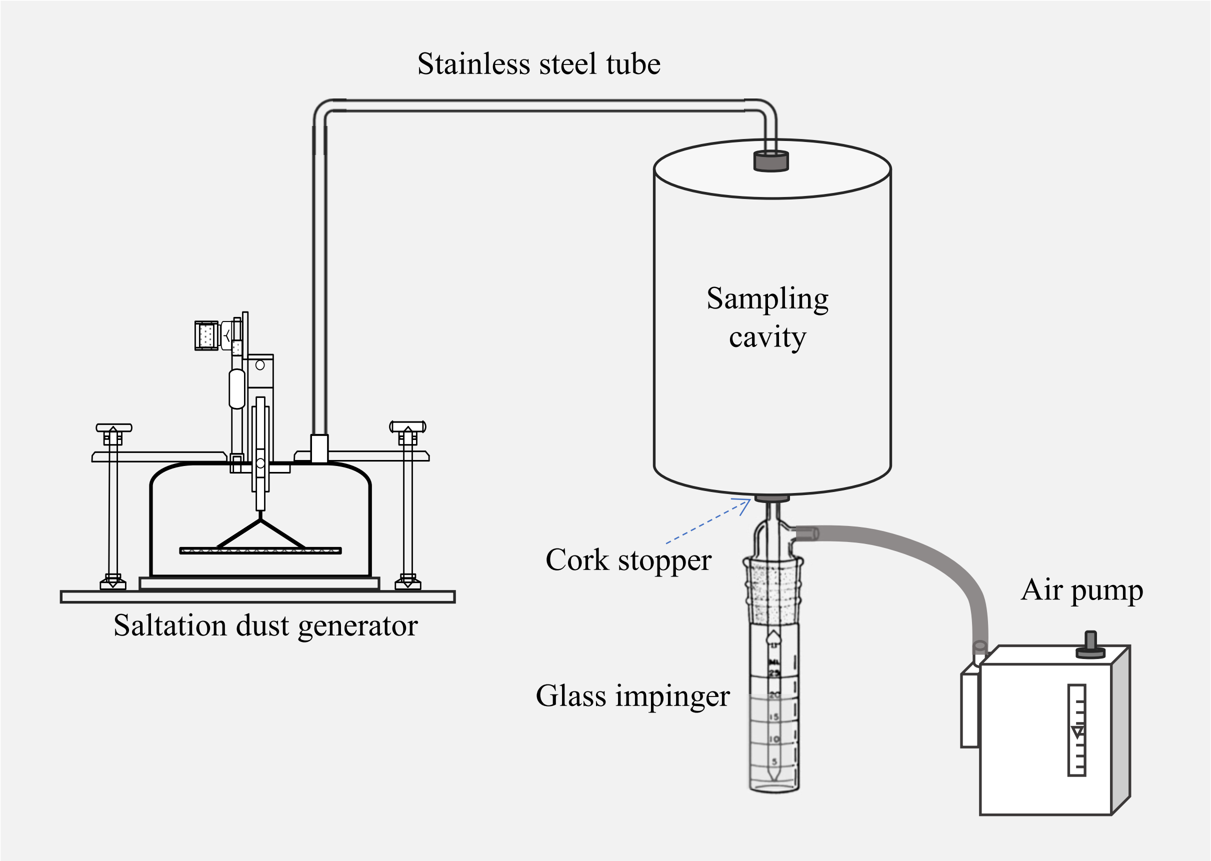

Fig. S11. Schematic of resuspension sampling.

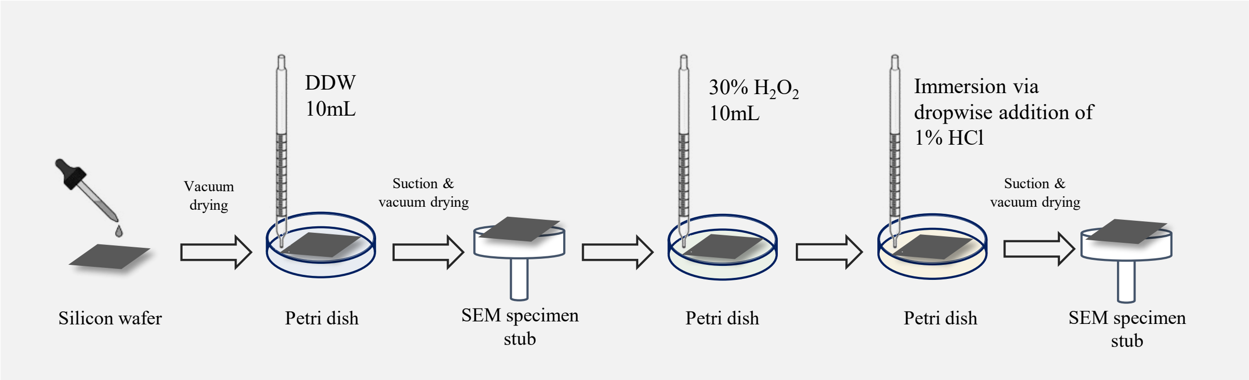

Fig. S12. Schematic of pretreatment procedures.

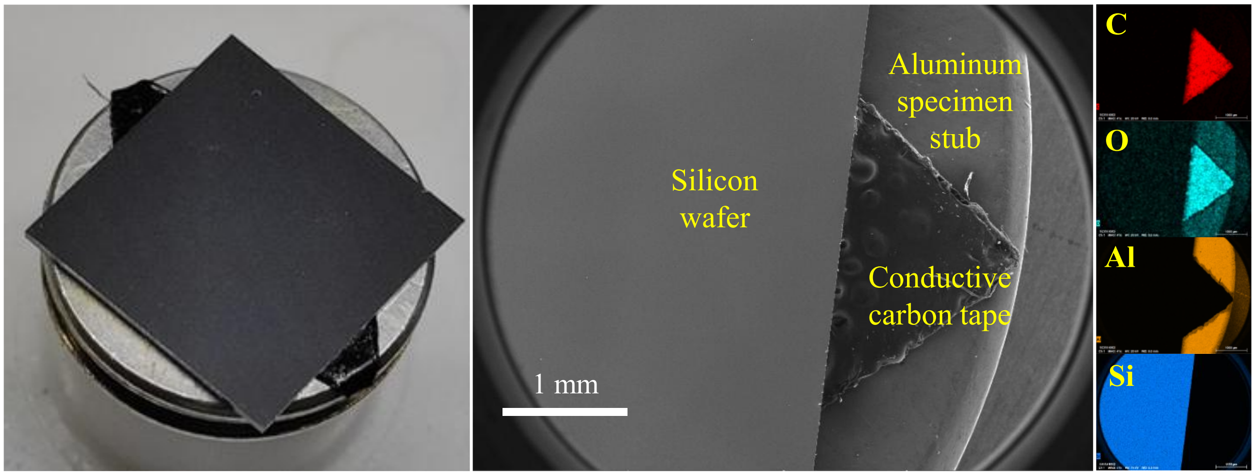

Fig. S13. SEM-EDX sample preparation protocol. Silicon wafer affixed to aluminum SEM specimen stub using double-sided conductive carbon tape. EDX elemental mapping showing spatially constrained distributions of substrate-related elements (C, O, Al) with negligible interference in particle analysis regions.

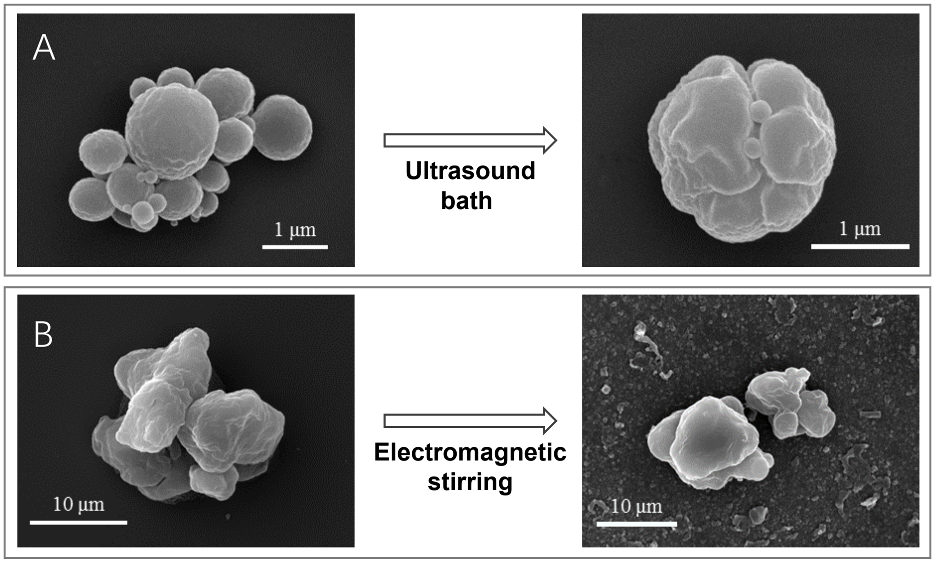

Fig. S14. Micrographs demonstrating ultrasonic- and electromagnetic stirring-induced breakdown and surface degradation of plastic reference materials. (**A**) Fracture development on the surface of PVC particles after 15-second ultrasound oscillation and (**B**) Fragmentation occurred on PE particles after 1-minute electromagnetic stirring. PVC, polyvinyl chloride. PE, polyethylene.

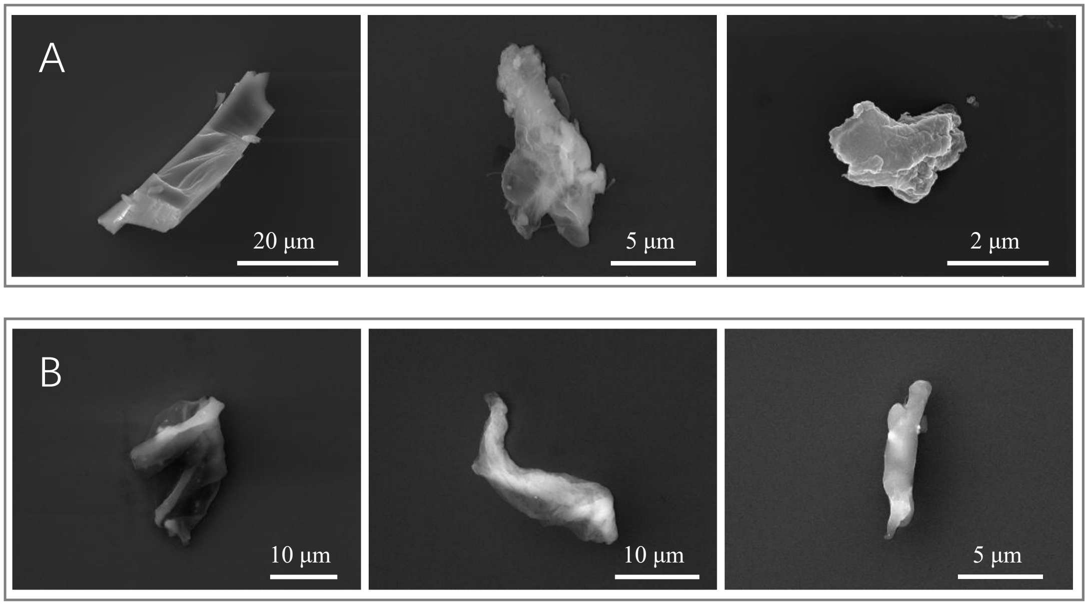

Fig. S15. Micrographs of particulate carbon contamination in solvents. (**A**) Deionized water, DIW and (**B**) Double-distilled water, DDW.

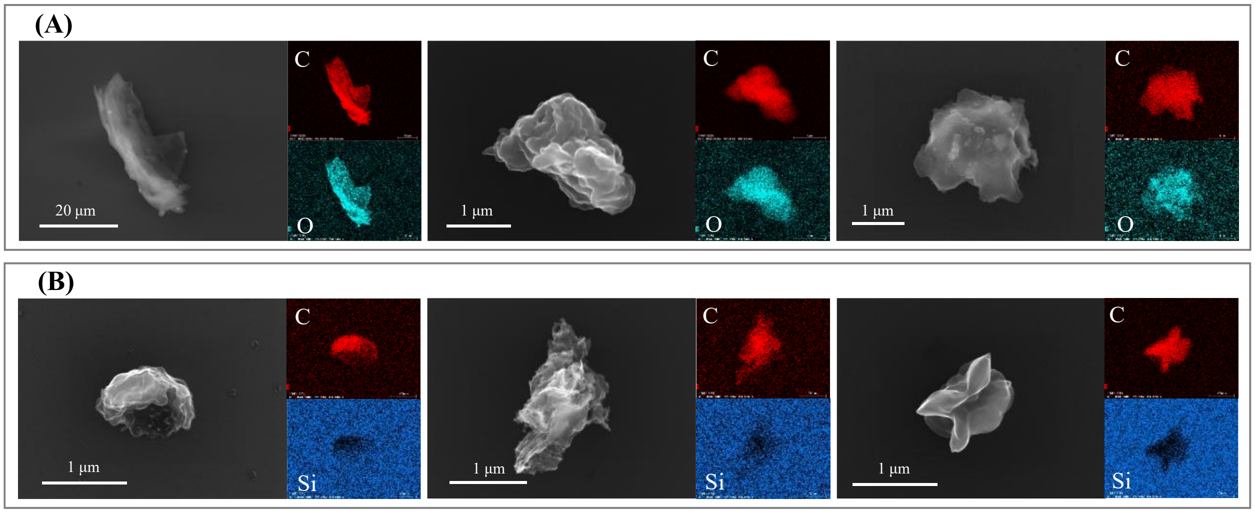

Fig. S16. Micrographs of particulate carbon contamination in reagents and their EDX elemental mappings. (**A**) hydrogen peroxide and (**B**) hydrochloric acid.

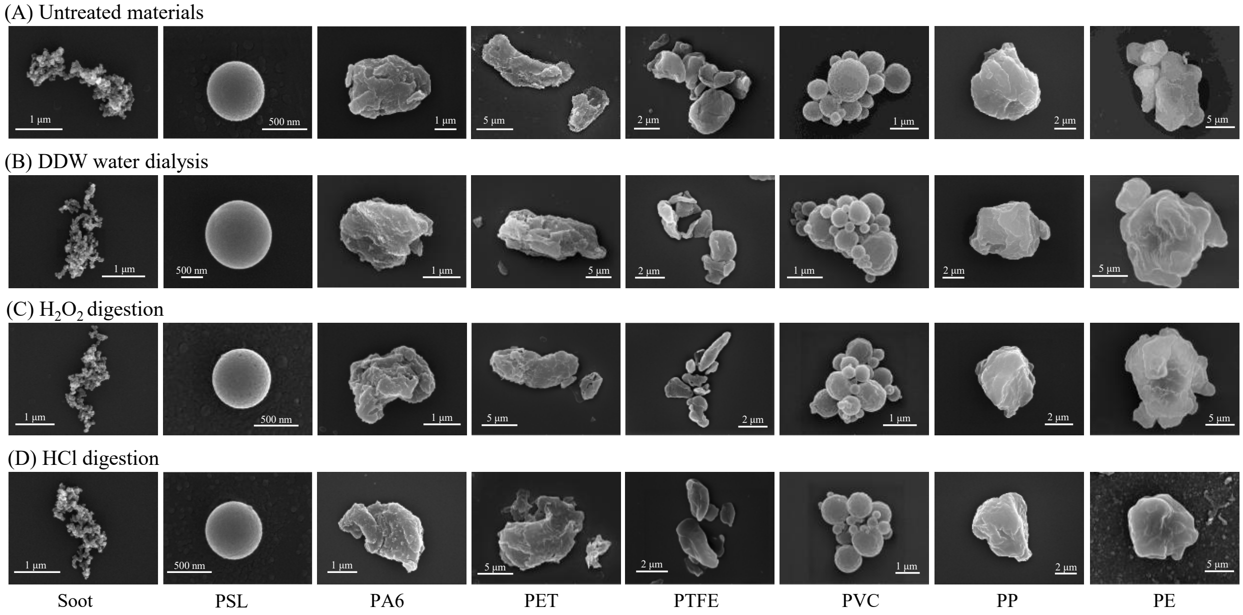

Fig. S17. Morphological evolution of reference materials across pretreatment stages. (**A**) Original morphologies, (**B**) Post-water dialysis, (**C**) Following subsequent H_2O_2 digestion and, (**D**) HCl digestion altered surface morphology of PE particles. PSL, polystyrene latex. PA6, polyamide 6. PET, polyethylene terephthalate. PP, polypropylene.

Table S1. Determination of CCSEM Detection Limit Using C_3N_4 Reference.

Items	D_p < 200 nm	D_p ≥ 200 nm
Number of particles rejected (LowEDS)	1325	181
Number of particles detected (C-rich)	215	2981
Typical images of C_3N_4 and EDX elemental mappings	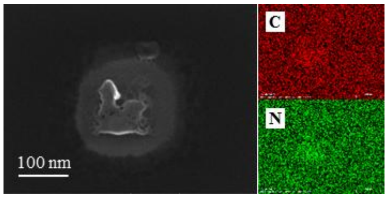	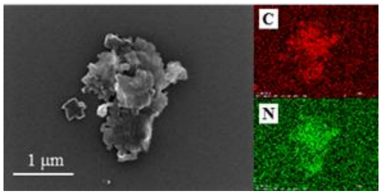

Note: The detection limit test used laboratory-synthesized C_3N_4 (from urea calcined in air) as a reference. A particle was rejected by the CCSEM system and marked as "LowEDS" when the signal-to-noise ratio (SNR) of its carbon and nitrogen EDX signals was below 3.

Table S2. Collection efficiency of impinger sampling for TSP samples.

Size range (μm)	Aerosol number concentration Average ± S.D. (Particles L^{-1})		Particle collection efficiency (%)
	Upstream of the impinger	Downstream of the impinger	
$0.30<D_p\leqslant 0.35$	129170±34997	34235±4559	73.5
$0.35<D_p\leqslant 0.40$	93044±25385	21327±2680	77.1
$0.40<D_p\leqslant 0.45$	57779±19615	11958±1613	79.3
$0.45<D_p\leqslant 0.50$	25049±9646	4296±594	82.9
$0.50<D_p\leqslant 0.60$	36843±12193	5001±733	86.4
$0.60<D_p\leqslant 0.70$	8021±2996	956±161	88.1
$0.70<D_p\leqslant 0.80$	4973±1603	497±86	90.0
$0.80<D_p\leqslant 1.00$	3753±850	352±53	90.6
$1.00<D_p\leqslant 1.30$	968±121	91±16	90.7
$1.30<D_p\leqslant 1.60$	471±42	38±7	91.8
$1.60<D_p\leqslant 2.00$	500±58	35±9	93.0
$2.00<D_p\leqslant 2.80$	300±44	12±4	96.0
$2.80<D_p\leqslant 5.00$	224±46	4±2	98.0
$5.00<D_p\leqslant 6.00$	36±11	0	100.0
$6.00<D_p\leqslant 8.00$	35±10	0	100.0
$8.00<D_p\leqslant 10.00$	17±8	0	100.0
$D_p>10.00$	11±7	0	100.0

Table S3. Normalized weight percentage of carbon and silicon from EDX point spectra at three random positions on the conductive silicon wafer under various magnifications.

Magnification	50×	500×	1000×	5000×	10000×	50000×
**C wt%**	5.23±0.07	5.34±0.07	5.31±0.04	5.35±0.10	5.29±0.02	5.55±0.14
**Si wt%**	94.77±0.07	94.66±0.07	94.69±0.04	94.65±0.10	94.71±0.02	94.45±0.14

Table S4. Particle classification criteria based on EDX-derived elemental weight percentages.

Classifications	Classification rules by EDX wt%	Potential materials
C-excluded	C < 10.0	mineral dust
C-rich	C ≥ 99.5	PP, PE, PS, rubber, etc.
C+O-rich	C < 99.5, C + O ≥ 99.5, O ≥ 0.5	PET, PC, PVA, PMMA, epoxy resin, etc.
C+others	C ≥ 10.0, (N + F + Al + S + Cl + Zn + Ti) ≥ 0.5	PVC, PTFE, PU, PA, or polymer with additives, etc.

Table S5. Precision of CCSEM for particle size and carbonaceous identification.

Repeatability	CCSEM measured PSL size (nm)	C_3N_4 in identical regions		C_3N_4 in separate regions	
		Detection rate (%)	Measured size (nm)	Detection rate (%)	Measured size (nm)
measurement 1	794 ± 16<br>(n = 6275)	93.6<br>(2799/2991)	838 ± 565<br>(n = 2799)	91.5<br>(2728/2983)	783 ± 539<br>(n = 2728)
measurement 2	796 ± 16<br>(n = 6485)	93.6<br>(2776/2965)	831 ± 538<br>(n = 2776)	92.5<br>(2717/2936)	731 ± 436<br>(n = 2717)
measurement 3	788 ± 15<br>(n = 6577)	91.8<br>(2659/2897)	807 ± 561<br>(n = 2659)	90.9<br>(2642/2905)	756 ± 519<br>(n = 2642)

Note: The nominal size of PSL reference is 802 nm ± 6 nm. The C_3N_4 reference is identical to Table 1. The numbers in the parentheses are the numbers of individual PSL sphere, identified C_3N_4 particles and total detected particles, shown as (n = sphere counts), (C-rich/total particles), and (n = C-rich particle counts), respectively.

Table S6. Spike recovery of reference materials and commercially available polymer micro-powders.

Reference materials	Manufacturer or supplier	Product name	Nominal D_p (μm)	CCSEM measured D_p (μm)	Morphology	Recovery rate (%)
BC	Degussa-Hüls	Printex U	with an average primary particle size of 27–31 nm	with a broad range of aggregation	aggregates of primary spheres	n.a.
PS	Duke Scientific Corp.	PSL	0.7	0.8±0.2	monodisperse spheres	98.9
PA6	Hunan Yuehua Chemical Co., Ltd.	n.a.	5.0	3.2±1.5	irregular, rough	96.7
PET	Dongguan Huachuang Plastic Products, Inc.	n.a.	10.0	3.4±2.4	irregular, rough	83.8
PTFE	DuPont	Zonyl	3.0	3.2±1.8	irregular, rough	88.7
PVC	Junzheng Energy & Chemical Group, Inc.	n.a.	0.8	1.8±0.9	spherical, aggregates	89.0
PP	Sinopec Group Co., Ltd	n.a.	6.5	7.3±4.4	irregular, smooth	82.7
PE	Dongguan Huachuang Plastic Products, Inc.	n.a.	5.0	13.5±7.5	irregular, smooth	93.1

Note: BC, also called carbon black, or soot; PS, polystyrene; PA6, polyamide-6; PET, polyethylene terephthalate; PTFE, polytetrafluoroethylene; PVC, polyvinyl chloride; PP, polypropylene; PE, polyethylene; n.a.: not available.

Table S7. Procedural blank contamination levels of MPs/NPs across environmental sample matrices.

Sample	MP							NP						
	unit	Xi'an			Guangzhou			unit	Xi'an			Guangzhou		
		C-rich	C+O	C+ others	C-rich	C+O	C+ others		C-rich	C+O	C+ others	C-rich	C+O	C+ others
TSP	$\times 10^4$ MP m^{-3}	1	1	1	1	1	1	$\times 10^4$ NP m^{-3}	n.d.	n.d.	n.d.	n.d.	n.d.	n.d.
Dustfall	$\times 10^4$ MP m^{-2} d^{-1}	n.d.	1	1	1	1	1	$\times 10^4$ NP m^{-2} d^{-1}	n.d.	n.d.	n.d.	n.d.	n.d.	n.d.
Rainwater	$\times 10^4$ MP L^{-1}	1	4	2	3	6	2	$\times 10^4$ NP L^{-1}	n.d.	n.d.	n.d.	n.d.	n.d.	n.d.
Snow	$\times 10^4$ MP L^{-1}	2	6	3	n.a.	n.a.	n.a.	$\times 10^4$ NP L^{-1}	n.d.	n.d.	n.d.	n.a.	n.a.	n.a.
Resuspension	$\times 10^4$ MP m^{-3}	1	8	6	2	11	5	$\times 10^4$ NP m^{-3}	1	n.d.	n.d.	n.d.	n.d.	n.d.

Note: n.d.: not detected; n.a.: not available.
